# Spontaneous tumor lysis syndrome in patients with solid tumors: a scoping review of the literature

**DOI:** 10.1007/s12032-023-02108-4

**Published:** 2023-07-11

**Authors:** Michail Papapanou, Anastasios E. Athanasopoulos, Eleni Georgiadi, Stefanos A. Maragkos, Michalis Liontos, Dimitrios C. Ziogas, Dimitrios Damaskos, Dimitrios Schizas

**Affiliations:** 1 Society of Junior Doctors, Athens, 15123 Greece; 2 Second Department of Obstetrics and Gynecology, Medical School, “Aretaieion Hospital”, National and Kapodistrian University of Athens, 76 Vas. Sofias Ave, Athens, 11528 Greece; 3 2nd Department of Radiology, Medical School, University General Hospital “Attikon”, National and Kapodistrian University of Athens, 1 Rimini Str, Haidari/Athens, 12462 Greece; 4 School of Medicine, National and Kapodistrian University of Athens, Athens, 11527 Greece; 5 Department of Clinical Therapeutics, Division of Oncology, National and Kapodistrian University of Athens, Alexandra Hospital, 80 Vas. Sofias Ave, Athens, 10679 Greece; 6 First Department of Internal Medicine, Unit of Medical Oncology, National and Kapodistrian University of Athens, Laikon General Hospital, 17 Agiou Thoma Str, Athens, 11527 Greece; 7 Department of Upper GI Surgery, Royal Infirmary of Edinburgh, Edinburgh, Scotland, UK; 8 First Department of Surgery, National and Kapodistrian University of Athens, Laikon General Hospital, 17 Agiou Thoma Str, Athens, 11527 Greece

**Keywords:** Tumor lysis syndrome, Spontaneous, Solid tumor, Metastasis, Rasburicase, Allopurinol

## Abstract

No systematic synthesis of all cases of spontaneous tumor lysis syndrome (STLS) in adult patients with solid tumors is available to date. Herein, we aim to recognize specific STLS characteristics and parameters related to a worse prognosis. We conducted a systematic search for randomized controlled trials, cohorts, case-control studies, and case reports. The primary endpoints were death and the need for renal replacement therapy (RRT) due to STLS. We estimated crude odds ratios (ORs) with 95% confidence intervals (95%CI) via univariate binary logistic regression. We included one cohort of 9 patients and 66 case reports of 71 patients [lung cancer 15(21.1%)]. Regarding the case reports, most patients [61(87.1%)] had metastatic disease [liver 46(75.4%)], developed acute kidney injury [59(83.1%)], needed RRT [25(37.3%)], and died due to STLS [36(55.4%)]. Metastatic disease, especially in the liver [*p* = 0.035; OR (95%CI): 9.88 (1.09, 89.29)] or lungs [*p* = 0.024; 14.00 (1.37, 142.89)], was significantly associated with STLS-related death compared to no metastasis. Cases resulting in death had a significantly higher probability of receiving rasburicase monotherapy than receiving no urate-lowering agents [*p* = 0.034; 5.33 (1.09, 26.61)], or the allopurinol-rasburicase combination [*p* = 0.023; 7.47 (1.40, 39.84)]. Patients receiving allopurinol were less likely to need RRT compared to those not receiving it or those receiving rasburicase. In conclusion, current anecdotal evidence demonstrated that metastatic disease, especially in the liver and lungs, may be associated with STLS-related death compared to no metastatic status. Careful surveillance of high-risk cases within larger studies is essential to identify markers predicting morbidity or mortality.

**Supplementary Information:**

The online version contains supplementary material available at 10.1007/s12032-023-02108-4.

## Introduction

Tumor lysis syndrome (TLS) is a common oncological emergency and is caused by the massive lysis of tumor cells mediated by anticancer therapies or spontaneous cellular death in rapidly dividing tumors [1, 2]. The rapid lysis leads to massive efflux of intracellular potassium, phosphorus, and uric acid into the circulation, and subsequent electrolyte abnormalities including hyperkalemia, hyperuricemia, hyperphosphatemia, and secondary hypocalcemia, that may require renal replacement therapy (RRT) to be recovered [1, 2]. TLS typically occurs in patients with leukemias (especially acute lymphoblastic leukemia), lymphomas (chiefly non-Hodgkin’s), and less commonly in cases with certain types of solid tumors, such as hepatoblastoma, neuroblastoma, and melanoma [1–4]. However, recent advances in oncological treatments have led to greater responses in high-burden solid tumors sensitive to cytotoxic agents and an increase in the incidence of TLS even in those rarely associated with this syndrome [1, 2, 4–6]. Despite this increase, the incidence of TLS in cases with solid tumors remains lower than in those with hematological malignancies [1, 2, 4].

In cases with solid tumors, the syndrome is usually induced by the administration of chemotherapeutic drug combinations, chemoembolization, radiotherapy, radiofrequency ablation (RFA), immune checkpoint inhibitors, monoclonal antibodies, or even corticosteroids - the latter more commonly reported in cases with hematological malignancies [3, 4, 6, 7]. Even less frequently, TLS may emerge in patients without recent exposure to any of the above therapeutic agents or approaches. In this setting, it is characterized as spontaneous TLS (STLS) [2, 8]; and more reports have linked the “STLS” in solid tumors with prior biopsies and oncological surgeries [8].

To date, the vast majority of available evidence on STLS is derived from hematological malignancies [3, 9] while in cases with solid tumors, TLS has been mainly attributed to previous oncological treatments [6, 10], and its spontaneous presentation is even less studied. This scoping review aims to explore the diagnostic characteristics, the clinical and laboratory presentation findings, as well as the management, and the prognosis of patients with solid tumors that developed STLS. In addition, any potential associations of examined variables with STLS-related death or hemodialysis will be investigated.

## Methods

### Study design and protocol registration

This scoping literature review was conducted in line with the extension for Scoping Reviews of the Preferred Reporting Items for Systematic Reviews and Meta-Analyses (PRISMA) [11]. The protocol was preregistered with Open Science Framework (OSF preregistration: 10.17605/OSF.IO/FWTZP, August 19, 2022).

### Eligibility criteria

Eligible were considered adult patients (> 18 years) with non-hematological/solid malignancies developing laboratory STLS (LSTLS) as per Cairo-Bishop criteria (i.e., at least two identified abnormalities in the serum concentrations of potassium, phosphorus, uric acid, and calcium) [12]. The laboratory definition of TLS was preferred to avoid the exclusion of initially asymptomatic cases [8]. Spontaneous cases were considered those without prior recent exposure to chemotherapy/radiation, locoregional anti-cancer treatments (e.g., chemoembolization, RFA), immune checkpoints inhibitors/monoclonal antibodies, and hormonal agents (in case of hormone-dependent cancers). After an explicit search of the relevant literature, we were unable to identify specific cut-off time points after the last administration of responsible agents that define the spontaneous character of the TLS. The Cairo-Bishop diagnostic criteria of non-spontaneous TLS generally require the presence of laboratory abnormalities within 3 days before or 7 days after the cytotoxic treatment [12]. Therefore, TLS was characterized as spontaneous only when laboratory diagnosis was confirmed at least one month after the last administration of the responsible cytotoxic agent. This interval was used to ensure the spontaneous nature of the syndrome even in case of bias associated with the retrospective design, expecting that most of the included literature would consist of anecdotal case reports/series [6, 13]. Incidents of TLS accompanied by recent prior exposure to invasive procedures like biopsies have been included. Although tumor biopsy has been linked with STLS in solid tumors on an anecdotal basis [6, 8], it is not clearly reported whether it is performed or not, even by relevant case reports (i.e., not reporting of a tumor biopsy cannot guarantee that was not performed). Cases of prior recent exposure to corticosteroids were excluded from the primary analyses and were only included in supplementary sensitivity analyses, considering that their potential for inducing TLS has been better described in the context of hematological malignancies rather than solid tumors [8].

We included studies that reported on parameters belonging to at least one of the 4 outcome axes:

- Tumor-related data [i.e., primary site, histological subtype, and grading, duration between the initial diagnosis and STLS emergence, treatment for the primary site (as a potential decrease in tumor burden), duration between last exposure to triggering factors and TLS diagnosis, stage of disease, tumor size (i.e., largest transverse dimension), metastatic disease (yes/no) and metastatic site(s)];
- STLS-related characteristics including potentially triggering agents (i.e., other than classic cytotoxic anticancer agents or previously described tumoricidal agents), clinical presentation [i.e., Cairo-Bishop grade of clinical STLS (CSTLS) [2, 12] divided into “no CSTLS but present LSTLS”, “mild CSTLS” (i.e., grade 1–2) and “severe CSTLS” (i.e., grade 3–4; patients that reached grade 5 were classified according to the grade of CSTLS on presentation, before dying due to the syndrome)], laboratory results at the time of diagnosis of LSTLS [e.g., urea/blood urea nitrogen (BUN), serum creatinine (SeCr), uric acid, potassium, phosphorus, calcium, sodium, white blood cell count (WBC), lactate dehydrogenase (LDH)], and subsequent complications (e.g., cardiac arrhythmia, new-onset seizures, acute kidney injury [2], and symptomatic hypocalcemia);
- Management of STLS and its related complications [i.e., administration of allopurinol, febuxostat, and/or rasburicase and other supportive medications (e.g., rehydration, and management of electrolyte imbalance)] [1, 2];
- Prognosis of STLS [i.e., need for RRT, case-fatality rate/death due to STLS, all-cause death, recurrence]. Death due to STLS (yes/no) was defined as death during admission for STLS and caused by the syndrome’s manifestations or direct complications.

We considered death or the need for RRT due to STLS as primary outcomes and the rest as secondary.

Published randomized controlled trials (RCTs), prospective/retrospective cohorts, case-control studies, case series, or case reports written in English, German, or French were considered eligible.

### Search strategy and study selection

A systematic literature search of the PubMed and Scopus databases and the CENTRAL registry (which contains the clinicaltrials.gov and WHO ICTRP registers) was conducted using the following algorithm: “Tumor Lysis Syndrome” AND spontaneous. A preliminary search was conducted on May 1, 2022. We updated the search up to October 17, 2022, to not exclude any recently published eligible studies. The obtained records were imported into EndNote 20 and underwent semiautomatic deduplication [14]. Deduplicated records were then imported into the Rayyan web application (https://www.rayyan.ai/). Three independent reviewers (EG, SAM, AEA) initially screened the records for relevance based on title and abstract only. Disagreements were resolved through consensus or by a fourth reviewer’s (MP) decision. Relevant articles were then assessed for eligibility by the same three independent reviewers with inconsistencies having been again addressed through discussion until consensus or decision by a fourth author (MP). References of the included articles were also searched, as per the snowball procedure, to further investigate cases not referred to as “spontaneous” by the authors. In case of overlapping/duplicate samples within the same analysis, the study with the most comprehensive report on the outcomes of interest was selected.

### Data items and data collection

Three blinded reviewers (EG, SAM, AEA) extracted data from the eligible articles in a predesigned Excel spreadsheet. Discrepancies were resolved through discussion until consensus. Items for which data were sought and extracted are presented in Supplementary methods.

### Risk of bias assessment

Within the scoping nature of this review, the risk of bias assessment of the eligible studies was not considered obligatory [11]. Since most eligible studies were expected to be case reports/case series, which are generally considered low-quality data [13], further risk of bias assessment was not performed.

### Statistical analysis

Reporting was separate for aggregate data provided by RCTs/observational studies and individual participant data (IPD) recorded by case reports. Regarding IPD, a secondary analysis of the collected data was performed. Numerical variables were presented as mean, standard deviation (SD), or median, [1st quartile 1 (Q1), 3rd quartile (Q3)] if their distributions were skewed, while categorical variables were presented as frequencies (%). The normality of continuous variables was examined by applying the Shapiro-Wilk test. Descriptive statistics of the examined variables were calculated for the overall sample and the sample of each one of the different primary tumor sites. Inferential statistics using the entire sample were restricted to the primary outcomes. Means of independent samples were compared using the independent-samples t-test if data were normally distributed; if not, the Mann-Whitney U Test was performed [15]. Chi-square/Fisher’s exact tests were employed for the comparisons between categorical data [16]. Crude odds ratios (ORs) along with their 95% confidence intervals (95%CI) and corresponding p-values (p_OR_) were estimated by univariate binary logistic regression with “death due to STLS”, and “need for RRT” serving as the dependent variables and one of the rest of the investigated variables being, each time, used as the predictor variable [17]. Multivariable logistic regression was not performed due to the small sample size [18, 19]. A supplementary sensitivity analysis, also including cases with recent prior exposure to corticosteroids, was carried out. All analyses were conducted with the Stata Statistical Software, version 13 (StataCorp LP, College Station, TX, USA). Two-tailed p-values less than 0.05 were considered statistically significant.

## Results

**Fig. 1:**
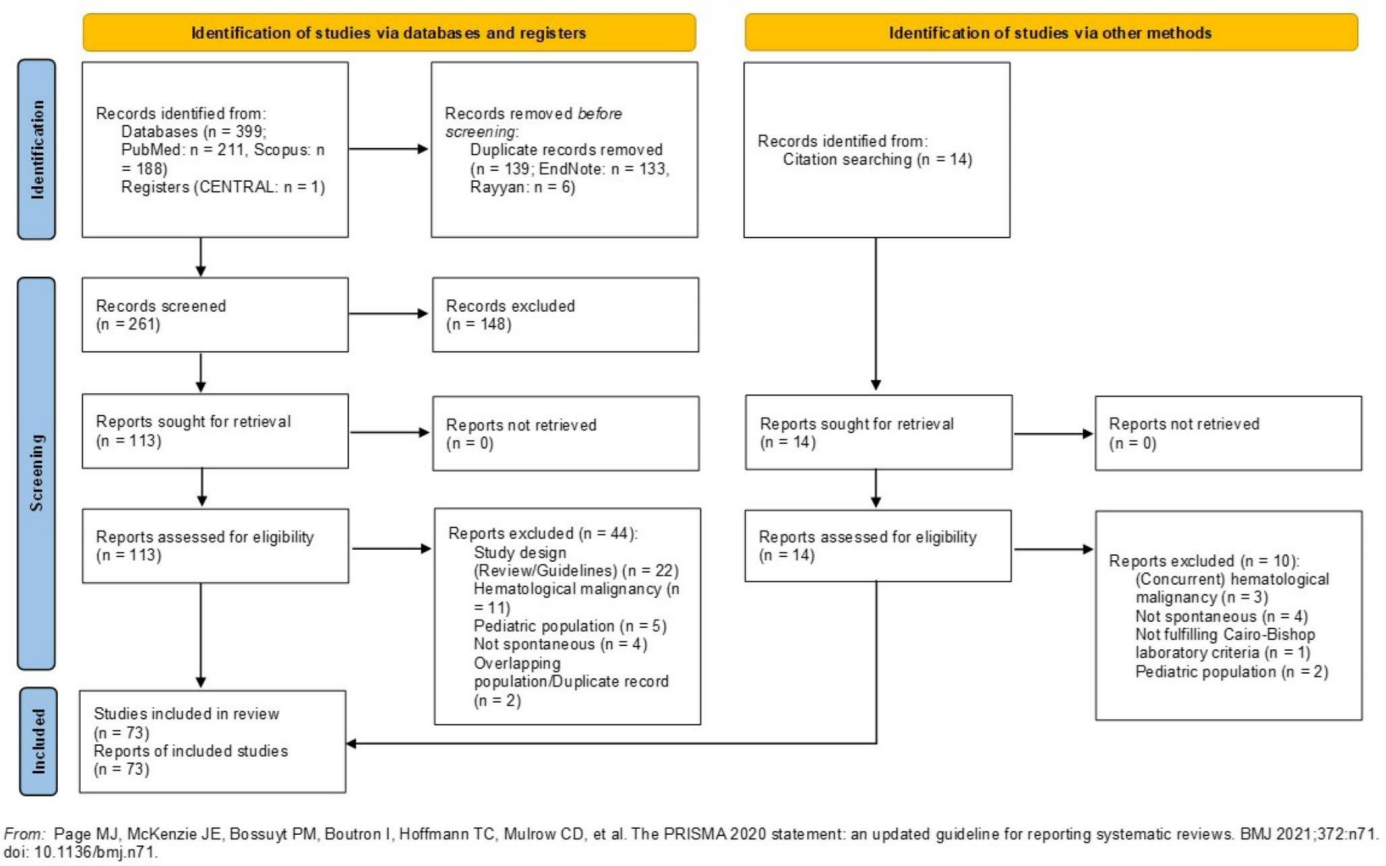
PRISMA flow diagram of the review

The literature search yielded 414 records. Following semiautomatic deduplication and title-abstract screening, 127 reports were assessed for eligibility. Of these, 54 were excluded and 73 (i.e., 71 case reports, 1 retrospective cohort with 9 patients eligible for inclusion [20], and 1 retrospective cohort with only 1 patient eligible for inclusion and thus regarded as case report [21]) were finally included in the review. Six case reports [22–27] considered cases of TLS after prior recent exposure to corticosteroids; these were only included in the supplementary analysis. Therefore, 67 studies [20, 21, 28–92] were included in the main analysis. The numbers of excluded studies as well as the exact reasons for their exclusion are available in Fig. 1 and Table S1, respectively.

The main characteristics of the included studies are presented in Table S2. The 66 case reports encompassed 71 patients. A minor overlap may likely exist between a cohort and a case report; [20, 58] however, these were not part of the same analysis and hence both were included. The demographic characteristics of individual patients, the primary tumor site and histology, the metastatic burden, the clinical and laboratory findings of developed STLS, as well as the management and prognosis of the syndrome, are available in Table 1, *and* Table 2. Same data is presented only for cases of TLS developing after administration of corticosteroids and for all cases, regardless of prior exposure to corticosteroids, in Table S3, S4, respectively. Variables are stratified according to primary tumor site in Table S5.

**Table 1:** Characteristics of included participants. Unless otherwise stated, categorical variables are presented as frequencies (%) and continuous variables are presented as median (first quartile, third quartile). Data on patients deriving from the case reports and those from the cohort are separately presented. Abbreviations: N, number of individuals whose data on the outcome was available; Q1, 1st quartile; Q3, 3rd quartile; SD, standard deviation; STLS, spontaneous tumor lysis syndrome; NA, not applicable; CSTLS, clinical spontaneous tumor lysis syndrome; LSTLS, laboratory spontaneous tumor lysis syndrome

Variable	Case reports		Cohort
	N	Values	
Total N	71		9
Age [median (Q1, Q3) / mean (SD)]	70	59.5 (49.0, 70.0)	63 (13)
Sex, male (%)	71	38 (53.5%)	8 (88.9%)
Charlson Comorbidity Index on admission	69	7 (6, 9)	7 (4, 9)
Classified Charlson Comorbidity Index on admission	69	Moderate: 3 (4.3%)	NA
		Severe: 66 (95.7%)	
**Tumor characteristics**			
Diagnosis of STLS along with the first diagnosis of the primary tumor	69	40 (58%)	NA
Time between diagnosis of the primary tumor and STLS (days)	58	5 (0, 34)	37 (12, 49)
Primary sites	71	Lung: 15 (21.1%)	Lung: 3 (33.3%)
		Skin: 3 (4.2%)	Stomach: 1 (11.1%)
		Colon: 6 (8.5%)	Colon: 1 (11.1%)
		Liver: 5 (7.0%)	NA
		Prostate: 3 (4.2%)	NA
		Uterus (Endometrium/Myometrium): 4 (5.6%)	Uterus (Endometrium): 1 (11.1%)
		Stomach: 4 (5.6%)	NA
		Breast: 3 (4.2%)	NA
		Kidney: 3 (4.2%)	NA
		Ovary: 2 (2.8%)	NA
		Pancreas: 2 (2.8%)	NA
		Adrenal gland: 2 (2.8%)	NA
		Uterus – cervix: 1 (1.4%)	NA
		Gallbladder: 1 (1.4%)	NA
		Esophagus: 1 (1.4%)	Esophagus: 1 (11.1%)
		Other: 10 (14.1%)	Other: 1 (11.1%)
		Unknown: 6 (8.5%)	Unknown: 1 (11.1%)
Tumor size (largest transverse diameter in cm)	30	8.9 (6, 13)	NA
Histological grade	26	Low: 8 (30.8%)	NA
		Intermediate: 2 (7.7%)	
		High: 16 (61.5%)	
Lymph nodes	35	No: 1 (2.9%)	NA
		Regional: 8 (22.8%)	
		Distal: 26 (74.3%)	
Metastasis	70	61 (87.1%)	NA
Metastatic sites	61	Liver: 21 (34.4%)	NA
		Lung: 6 (9.9%)	
		Bones: 2 (3.3%)	
		Liver & lung: 12 (19.7%)	
		Liver & bones: 10 (16.4%)	
		Lung & bones: 1 (1.6%)	
		Liver, lung & bones: 3 (4.9%)	
		No liver, lung, or bones (but other metastatic sites): 5 (8.2%)	
		Unknown (disease presented as metastatic but sites not reported): 1 (1.6%)	
Stage	68	I: 1 (1.5%)	NA
		II: 1 (1.5%)	
		III: 5 (7.3%)	
		IV: 61 (89.7%)	
Had received any kind of treatment for the primary tumor before the STLS diagnosis	69	12 (17.4%)	NA
Had received surgical treatment for primary tumor before STLS diagnosis	69	8 (11.6%)	NA
Had received any kind of treatment for metastatic tumor before the STLS diagnosis	60	1 (1.7%)	NA
Had received surgical treatment for metastatic tumor before STLS diagnosis	60	1 (1.7%)	NA
Time between diagnosis of metastatic tumor(s) and STLS (days)	57	0 (0, 6)	NA
**STLS characteristics**			
Acute kidney injury	71	59 (83.1%)	9 (100%)
Cardiac arrhythmia	71	3 (4.2%)	3 (33.3%)
New-onset seizure	71	1 (1.4%)	5 (55.6%)
Symptomatic hypocalcemia	71	2 (2.8%)	1 (11.1%)
Blood urea nitrogen (mg/dl)	39	73.7 (54, 100)	NA
Serum creatinine (mg/dl) [median (Q1, Q3) / mean (SD)]	64	3.5 (2.1, 4.7)	2.9 (1.5)
Uric acid (mg/dl) [median (Q1, Q3) / mean (SD)]	67	15.0 (12.8, 20.3)	16.3 (5.0)
Potassium (mmol/l) [mean (SD)]	62	6.0 (0.9)	6.2 (1.0)
Phosphorus (mg/dl) [median (Q1, Q3) / mean (SD)]	67	6.8 (5.2, 8.6)	7.3 (3.3)
Calcium (mg/dl) [mean (SD)]	55	8.2 (1.6)	8.3 (0.8)
Sodium (mmol/l)	22	132 (126, 139)	NA
Lactate dehydrogenase (U/l) [median (Q1, Q3) / mean (SD)]	47	1 449 (864, 3922)	1 554 (1 010)
White blood cells (/µL)	23	16 950 (12 300, 30 300)	NA
Cairo-Bishop clinical grade on admission	66	No CSTLS but LSTLS: 7 (10.6%)	
		1: 12 (18.2%)	NA
		2: 20 (30.3%)	
		3: 23 (34.8%)	
		4: 4 (6.1%)	
Cairo-Bishop clinical grade on admission (no or mild vs. severe)	66	No or Mild CSTLS: 39 (59.1%)	NA
		Severe CSTLS: 27 (40.9%)	
Cairo-Bishop clinical grade on admission (mild vs. severe)	59	Mild CSTLS: 32 (54.2%)	NA
		Severe CSTLS: 27 (45.8%)	
**Management of STLS**			
Received allopurinol	66	32 (48.5%)	NA
Received febuxostat	65	1 (1.5%)	NA
Received allopurinol/febuxostat	66	33 (50.0%)	NA
Received rasburicase	65	33 (50.8%)	NA
Received allopurinol/febuxostat/rasburicase	66	51 (77.3%)	NA
Received allopurinol/febuxostat and rasburicase	65	15 (23.1%)	NA
Received allopurinol and rasburicase	65	14 (21.5%)	NA
Received allopurinol and febuxostat	65	0 (0%)	NA
Received febuxostat and rasburicase	65	1 (1.5%)	NA
Type of urate-lowering treatment	66	Allopurinol monotherapy: 18 (27.3%)	NA
		Febuxostat monotherapy: 0 (0%)	
		Rasburicase monotherapy: 18 (27.3%)	
		Allopurinol & Rasburicase: 14 (21.2%)	
		Febuxostat & Rasburicase: 1 (1.5%)	
		No urate-lowering treatment: 15 (22.7%)	
Received insulin	64	8 (12.5%)	NA
Received calcium gluconate	64	4 (6.3%)	NA
Needed/received renal replacement therapy	67	25 (37.3%)	0 (0%)
**Prognosis of STLS**			
Death related to STLS	65	36 (55.4%)	7 (77.8%)
All-cause death	65	52 (80.0%)	NA
Time between diagnosis of STLS and death (days)	38	4 (1, 10)	NA
Discharge	52	18 (34.6%)	NA
Time between diagnosis of STLS and discharge (days)	9	11 (10, 14)	NA
Recurrence	70	2 (2.9%)	NA

**Table 2:** Histological types and metastatic sites by primary tumor site. Categorical variables are presented as frequencies (%)

Study type, N	Primary tumor site	Histological types per primary tumor site	Metastatic sites per primary tumor site
Case reports, N = 71	Lung: 15 (21.1%)	Squamous carcinoma: 5 Adenocarcinoma: 1 Small cell lung cancer: 8 Unknown: 1	Liver: 9 Bones: 1 Liver & lungs: 1 Liver & bones: 1 Unknown: 1 No metastasis: 2
	Skin: 3 (4.2%)	Melanoma: 3	Liver & lungs: 1 Liver, lung & bones: 1 Other: 1
	Colon: 6 (8.5%)	Adenocarcinoma: 6	Liver: 3 Lung: 1 Liver & lung: 1 Liver & bones: 1
	Liver: 5 (7.0%)	Hepatocellular carcinoma: 3 Adenocarcinoma: 1 Unknown: 1	Lung: 3 No metastasis: 1 Unknown: 1
	Prostate: 3 (4.2%)	Adenocarcinoma: 3	Bones: 1 Liver & bones: 2
	Uterus (Endometrium/Myometrium): 4 (5.6%)	Endometrial adenocarcinoma: 2 Leiomyosarcoma: 1 Neuroendocrine carcinoma: 1	Liver: 1 Lung: 1 No metastasis: 2
	Stomach: 4 (5.6%)	Adenocarcinoma: 4	Liver: 2 Liver & bones: 1 Other: 1
	Breast: 3 (4.2%)	Adenocarcinoma: 3	Liver & lung: 1 Liver, lung & bones: 1 No metastasis: 1
	Kidney: 3 (4.2%)	Adenocarcinoma: 1 Sarcoma: 1 Urothelial carcinoma: 1	Liver & bones: 2 No metastasis: 1
	Ovary: 2 (2.8%)	Adenocarcinoma: 2	Other: 1 No metastasis: 1
	Pancreas: 2 (2.8%)	Adenocarcinoma: 2	Liver: 1 Liver & lung: 1
	Adrenal gland: 2 (2.8%)	Adenocarcinoma: 1 Pheochromocytoma: 1	Liver: 1 No metastasis: 1
	Uterus – cervix: 1 (1.4%)	Squamous carcinoma: 1	Lung & bones: 1
	Gallbladder: 1 (1.4%)	Adenocarcinoma: 1	Liver: 1
	Esophagus: 1 (1.4%)	Squamous carcinoma: 1	Liver & bones: 1
	Other: 10 (14.1%)	Melanoma of the eyeball: 1	Liver: 1
		Retroperitoneal: 1 adenocarcinoma, 1 sarcoma, 1 seminoma, 1 choriocarcinoma	Liver & lung: 3 Unknown: 1
		Neck: 1 sarcoma	Liver & lung: 1
		Pelvis: 1 sarcoma, 1 primitive neuroectodermal tumor	Lung: 1 Liver & bones: 1
		Unknown paracaval mass	Liver & lung: 1
		Neuroblastoma in the upper left hemithorax: 1	Liver, lung & bones: 1
	Unknown: 6 (8.5%)	Adenocarcinoma: 4 Neuroendocrine carcinoma: 1 Unknown: 1	Liver: 2 Liver & lung: 2 Liver & bones: 1 Other: 1
Cohort, N = 9	Lung: 3 (33.3%)	Small cell lung cancer: 2 Adenocarcinoma: 1	NA
	Stomach: 1 (11.1%)	Adenocarcinoma: 1	NA
	Colon: 1 (11.1%)	Adenocarcinoma: 1	NA
	Uterus (Endometrium): 1 (11.1%)	Adenocarcinoma: 1	NA
	Esophagus: 1 (11.1%)	Squamous carcinoma: 1	NA
	Other: 1 (11.1%)	Quadriceps myxoid liposarcoma: 1	NA
	Unknown: 1 (11.1%)	NA	NA

Regarding the cohort’s nine patients [8 (89%) male, mean (SD) age of 63 (13), median (Q1, Q3) Charlson Comorbidity Index (CCI) on the admission of 7 (4, 9)], three (33.3%) had a primary lung tumor while the rest was diagnosed with gastric, colon and endometrial adenocarcinomas, esophageal squamous cell carcinoma, quadriceps myxoid liposarcoma and a tumor of unknown primary site [20]. AKI was reported in all patients, while new-onset seizures [5 (55.6%)] and cardiac arrhythmias [3 (33.3%)] were also frequent. Patients had an unfavorable course, with seven (77.8%) dying due to the syndrome and its related complications (Table 1) [20].

Τhe most common primary tumor sites in individually reported patients [38 (53.5%) male, median (Q1, Q3) age of 59.5 (49.0, 70.0), and CCI on admission of 7 (6, 9)] were lung [15 (21.1%)], colon [6 (8.5%)], and liver [5 (7.0%)]. The authors suggested increased age, large bulky masses [median (Q1, Q3) largest transverse dimension of 8.9 cm (6.0, 13.0)], metastatic disease, recent biopsy, dehydration, infection, preexisting kidney dysfunction or urinary obstruction, use of nephrotoxic drugs or medications that inhibit uric acid excretion, and increased baseline levels of WBC, phosphorus, potassium, uric acid or LDH as predisposing factors for the spontaneous emergence of TLS in adults with solid tumors. Forty (58%) patients had concurrent diagnosis of STLS and their underlying tumor. Most patients [61 (87.1%)] had developed metastatic disease, with the liver [46 (75.4%)] being the most reported site, followed by lung [22 (36.1%)] and bone involvement [16 (26.2%)]. At the time of presentation, seven (10.6%) had LSTLS without CSTLS, 32 (48.5%) had CSTLS which was classified as mild as per Cairo-Bishop grading [i.e., 12 (18.2%) grade 1 and 20 (30.3%) grade 2], and 27 [i.e., 23 (34.8%) grade 3 and 4 (6.1%) grade 4] had severe CSTLS. STLS resulted in AKI in the majority [59 (83.1%)] of individuals, while arrhythmias, seizures, and symptomatic hypocalcemia were prevalent in only three (4.2%), one (1.4%), and two (2.8%) cases, respectively. For the reduction of uric acid levels, most patients were administered either allopurinol [18 (27.3%)] or rasburicase monotherapy [18 (27.3%)], whilst the rest received no treatment [15 (22.7%)], or combinations of rasburicase with allopurinol [14 (21.2%)] or febuxostat [1 (1.5%)]. Patients had generally an unfavorable prognosis with high rates of need for RRT [25 (37.3%)], death related to STLS [36 (55.4%)], and all-cause death [52 (80.0%)]. Recurrence was rare (2.9%), with only 2 relevant incidents being recorded (Tables 1 and 2).

Patients with metastatic disease were significantly more likely to die due to STLS compared to those without any diagnosed metastasis [test of association *p* = 0.040; crude OR, 8.87; 95%CI, 1.001 to 78.63, *p*_*OR*_ = 0.049], especially when one of the metastatic sites was the liver [*p* = 0.035; OR, 9.88; 95%CI, 1.09 to 89.29, *p*_*OR*_ = 0.041] or lungs [*p* = 0.024; OR, 14.00; 95%CI, 1.37 to 142.89, *p*_*OR*_ = 0.026]. The same applied to individuals diagnosed with metastases only in the liver [OR, 19.20; 95%CI, 1.84 to 199.94, *p*_*OR*_ = 0.013] or lungs [OR, 24.00; 95%CI, 1.14 to 505.20, *p*_*OR*_ = 0.041] using again as reference the patient group without any diagnosed metastasis. Cases that received rasburicase monotherapy had also a significantly higher probability of resulting in death due to STLS than those receiving no urate-lowering treatment [*p* = 0.034; OR, 5.33; 95%CI, 1.09 to 26.61, *p*_*OR*_ = 0.041], or the allopurinol-rasburicase combination [*p* = 0.023; OR, 7.47; 95%CI, 1.40 to 39.84, *p*_*OR*_ = 0.019] (Table 3).

**Table 3:** Unified analyses on patients with solid tumors developing spontaneous tumor lysis syndrome (STLS). Characteristics of individuals resulting in death compared to those not resulting in death related to the STLS and its complications. Continuous data are presented as mean ± SD or median (1st quartile, 3rd quartile). All laboratory markers were measured at the point of the first establishment of the laboratory STLS. “Yes” refers to those exposed to the factor (1st column) whereas “No” to those not. Abbreviations: N, number of individuals whose data on the outcome was available; OR, odds ratio; CI, confidence interval; STLS, spontaneous tumor lysis syndrome; NA, not applicable; LDH, lactate dehydrogenase; WBC, white blood cells; ALL, allopurinol; FEB, febuxostat; RASB, rasburicase; RRT, renal replacement therapy

Factor	N	Overall	Death due to STLS	No death due to STLS	p-value	Univariate logistic regression, Crude OR (95% CI); p-value
Age	64	59 (49, 68.5)	59 (49, 69)	57 (51, 65)	0.792	1.00 (0.97, 1.03); p = 0.947
Sex (female vs. male)	65	Female: 31/65 Male: 34/65	Female: 16/31 Male: 20/34	Female: 15/31 Male: 14/34	0.559	0.75 (0.28, 1.99); p = 0.560
Charlson Comorbidity Index on admission	69	7 (6, 9)	8 (6, 8)	7 (6, 9)	0.688	1.05 (0.82, 1.33); p = 0.698
Diagnosis of STLS along with the first diagnosis of the primary tumor	63	Yes: 37/63 No: 26/63	Yes: 18/37 No: 17/26	Yes: 19/37 No: 9/26	0.188	0.50 (0.18, 1.41); p = 0.191
Time between diagnosis of the primary tumor and STLS (days)	53	5 (0, 34)	5.5 (1.5, 25.5)	4 (0, 210)	0.764	0.997 (0.993, 1.001); p = 0.131
Had received any kind of treatment for the primary tumor before the STLS diagnosis	63	Yes: 12/63 No: 51/63	Yes: 6/12 No: 29/51	Yes: 6/12 No: 22/51	0.667	0.76 (0.22, 2.67); p = 0.667
Had received surgical treatment for primary tumor before STLS diagnosis	63	Yes: 8/63 No: 55/63	Yes: 4/8 No: 31/55	Yes: 4/8 No: 24/55	1.000	0.77 (0.18, 3.42); p = 0.735
Stage	64	I: 1/64 II: 1/64 III: 5/64 IV: 57/64	I: 0/1 II: 0/1 III: 1/5 IV: 34/57	I: 1/1 II: 1/1 III: 4/5 IV: 23/57	0.077	(with III as a reference) IV: 5.91 (0.62, 56.34); p = 0.122
Tumor size	28	8.85 (6, 12)	10 (6, 13)	8 (4, 10.5)	0.549	1.03 (0.89, 1.20); p = 0.676
Lymph nodes	33	No: 1/33 Regional: 8/33 Distal: 24/33	No: 0/1 Regional: 4/8 Distal: 12/24	No: 1/1 Regional: 4/8 Distal: 12/24	1.000	(with “regional” as a reference) Distal: 1.00 (0.20, 4.95); p = 1.000
Metastasis	64	Yes: 57/64 No: 7/64	Yes: 34/57 No: 1/7	Yes: 23/57 No: 6/7	**0.040**	**8.87 (1.001, 78.63); p = 0.049**
Metastatic site(s)	64	Liver: 21/64 Lung: 5/64 Bones: 1/64 Liver & lungs: 11/64 Liver & bones: 10/64 Lung & bones: 1/64 Liver, lung & bones: 3/64 Metastatic but no liver, lung, or bones: 5/64 No metastasis: 7/64	Liver: 16/21 Lung: 4/5 Bones: 0/1 Liver & lungs: 7/11 Liver & bones: 3/10 Lung & bones: 1/1 Liver, lung & bones: 2/3 Metastatic but no liver, lung, or bones: 1/5 No metastasis: 1/7	Liver: 5/21 Lung: 1/5 Bones: 1/1 Liver & lungs: 4/11 Liver & bones: 7/10 Lung & bones: 0/1 Liver, lung & bones: 1/3 Metastatic but no liver, lung, or bones: 4/5 No metastasis: 6/7	**0.013**	(with “no metastasis” as a reference): Liver: **19.20 (1.84, 199.94); p = 0.013** Lungs: **24.00 (1.14, 505.20); p = 0.041** Liver & Lungs: 10.50 (0.91, 121.39); p = 0.060 Liver & Bones: 2.57 (0.21, 31.71); p = 0.461 Liver, lungs & bones: 12.00 (0.49, 294.57); p = 0.128 Metastatic but no liver, lung, or bones: 1.50 (0.07, 31.57); p = 0.794
Liver (as one of the metastatic sites) vs. No metastasis at all	52	Liver: 45/52 No metastasis: 7/52	Liver: 28/45 No metastasis: 1/7	Liver: 17/45 No metastasis: 6/7	**0.035**	**9.88 (1.09, 89.29); p = 0.041**
Lungs (as one of the metastatic sites) vs. No metastasis at all	27	Lung: 20/27 No metastasis: 7/29	Lung: 14/20 No metastasis: 1/7	Lung: 6/20 No metastasis: 6/7	**0.024**	**14.00 (1.37, 142.89); p = 0.026**
Bones (as one of the metastatic sites) vs. No metastasis at all	22	Bones: 15/22 No metastasis: 7/22	Bones: 6/15 No metastasis: 1/7	Bones: 9/15 No metastasis: 6/7	0.350	4.00 (0.38, 42.18); p = 0.249
Liver (as one of the metastatic sites) vs. No liver metastasis	63	Yes: 45/63 No: 18/63	Yes: 28/45 No: 7/18	Yes: 17/45 No: 11/18	0.092	2.59 (0.84, 7.96); p = 0.097
Lung (as one of the metastatic sites) vs. No lung metastasis	63	Yes: 20/63 No: 43/63	Yes: 14/20 No: 21/43	Yes: 6/20 No: 22/43	0.116	2.44 (0.79, 7.55); p = 0.120
Bones (as one of the metastatic sites) vs. No bone metastasis	63	Yes: 15/63 No: 48/63	Yes: 6/15 No: 29/48	Yes: 9/15 No: 19/48	0.165	0.44 (0.13, 1.43); p = 0.170
Time between STLS diagnosis and metastasis (days)	53	0 (0, 6)	0 (0, 6)	0 (0, 3)	0.575	0.996 (0.988, 1.004); p = 0.356
Had received any kind of treatment for metastatic tumor before the STLS diagnosis	57	Yes: 1/57 No: 56/57	Yes: 1/1 No: 32/56	Yes: 0/1 No: 24/56	1.000	Ν/Α
Had received surgical treatment for metastatic tumor before STLS diagnosis	57	Yes: 1/57 No: 56/57	Yes: 1/1 No: 32/56	Yes: 0/1 No: 24/56	1.000	Ν/Α
Cairo Bishop clinical STLS severity on presentation	61	Mild or No Clinical STLS: 36/61 Severe Clinical STLS: 25/61	Mild or No Clinical STLS: 20/36 Severe Clinical STLS: 13/25	Mild or No Clinical STLS: 16/36 Severe Clinical STLS: 12/25	0.784	0.87 (0.31, 2.41); p = 0.784
Cairo Bishop clinical STLS severity on presentation	55	Mild Clinical STLS: 30/55 Severe Clinical STLS: 25/55	Mild Clinical STLS: 17/30 Severe Clinical STLS: 13/25	Mild Clinical STLS: 13/30 Severe Clinical STLS: 12/25	0.729	0.83 (0.29, 2.41); p = 0.729
Acute kidney injury	65	Yes: 54/65 No: 11/65	Yes: 30/54 No: 6/11	Yes: 24/54 No: 5/11	1.000	1.04 (0.28, 3.83); p = 0.951
Cardiac arrhythmia	65	Yes: 3/65 No: 62/65	Yes: 2/3 No: 34/62	Yes: 1/3 No: 28/62	1.000	1.65 (0.14, 19.13); p = 0.690
New-onset seizure	65	Yes: 1/65 No: 64/65	Yes: 0/1 No: 36/64	Yes: 1/1 No: 28/64	0.446	N/A
Symptomatic hypocalcemia	65	Yes: 2/65 No: 63/65	Yes: 1/2 No: 35/63	Yes: 1/2 No: 28/63	1.000	0.80 (0.05, 13.37); p = 0.877
Blood urea nitrogen (mg/dl)	37	73.74 (54, 94)	76.5 (64.7, 100.2)	58.8 (45.92, 94)	0.152	1.01 (0.99, 1.03); p = 0.253
Serum creatinine (mg/dl)	59	3.5 (2.1, 4.7)	3.5 (2.1, 4.7)	3.50 (1.7, 4.9)	0.952	0.99 (0.80, 1.24); p = 0.939
Uric acid (mg/dl)	62	15.0 (13.1, 20.3)	15.8 (14.1, 20.3)	14.4 (10.7, 21.1)	0.198	1.05 (0.95, 1.16); p = 0.336
Potassium (mmol/l)	57	6.02 ± 0.94	6.03 ± 0.77	6.02 ± 1.18	0.982	1.01 (0.57, 1.79); p = 0.981
Phosphorus (mg/dl)	62	6.90 (5.26, 8.70)	7.10 (5.20, 9.20)	6.80 (5.26, 8.56)	0.707	1.05 (0.91, 1.21); p = 0.484
Calcium (mg/dl)	52	8.22 ± 1.60	8.34 ± 1.44	8.04 ± 1.83	0.501	1.13 (0.79, 1.61); p = 0.493
Sodium (mmol/l)	24	131.5 (125.5, 138.2)	129 (123, 133)	134 (127, 140)	0.202	0.93 (0.84, 1.04); p = 0.186
LDH (U/l)	43	1 470 (847, 4 023)	2 092 (992, 4 055)	1 203 (597, 3 163.5)	0.342	1.00 (0.99, 1.00); p = 0.867
WBC (/µl)	21	16 950 (13 800, 30 300)	20 500 (16 000, 32 000)	14 050 (11 660, 30 300)	0.121	1.00 (0.99, 1.00); p = 0.257
RASB vs. No RASB	61	Yes: 31/61 No: 30/61	Yes: 19/31 No: 16/30	Yes: 12/31 No: 14/30	0.530	1.39 (0.50, 3.84); p = 0.530
ALL vs. No ALL	62	Yes: 29/62 No: 33/62	Yes: 14/29 No: 21/33	Yes: 15/29 No: 12/33	0.224	0.53 (0.19, 1.47); p = 0.226
FEB vs. No FEB	61	Yes: 1/61 No: 60/61	Yes: 0/1 No: 35/60	Yes: 1/1 No: 25/60	0.426	N/A
At least 1 of ALL/FEB vs. No ALL/FEB	62	Yes: 30/62 No: 32/62	Yes: 14/30 No: 21/32	Yes: 16/30 No: 11/32	0.132	0.46 (0.16, 1.27); p = 0.135
At least 1 of ALL/FEB/RASB vs. No ALL/FEB/RASB	62	Yes: 47/62 No: 15/62	Yes: 28/47 No: 7/15	Yes: 19/47 No: 8/15	0.380	1.68 (0.52, 5.43); p = 0.382
ALL/FEB + RASB vs. No ALL/FEB + RASB	61	Yes: 14/61 No: 47/61	Yes: 5/14 No: 30/47	Yes: 9/14 No: 17/47	0.062	0.31 (0.09, 1.09); p = 0.069
ALL + RASB vs. No ALL + RASB	61	Yes: 13/61 No: 48/61	Yes: 5/13 No: 30/48	Yes: 8/13 No: 18/48	0.120	0.38 (0.11, 1.32); p = 0.127
FEB + RASB vs. No FEB + RASB	61	Yes: 1/61 No: 60/61	Yes: 0/1 No: 35/60	Yes: 1/1 No: 25/60	0.426	N/A
Urate-lowering treatment type	62	None: 15/62 ALL: 16/62 RASB: 17/62 ALL + RASB: 13/62 FEB + RASB: 1/62	None: 7/15 ALL: 9/16 RASB: 14/17 ALL + RASB: 5/13 FEB + RASB: 0/1	None: 8/15 ALL: 7/16 RASB: 3/17 ALL + RASB: 8/13 FEB + RASB: 1/1	0.062	See breakdown
ALL vs. None	31	ALL: 16/31 None: 15/31	ALL: 9/16 None: 7/15	ALL: 7/16 None: 8/15	0.594	1.47 (0.36, 6.05); p = 0.594
RASB vs. None	32	RASB: 17/32 None: 15/32	RASB: 14/17 None: 7/15	RASB: 3/17 None: 8/15	**0.034**	**5.33 (1.09, 26.61); p = 0.041**
ALL + RASB vs. None	28	ALL + RASB: 13/28 None: 15/28	ALL + RASB: 5/13 None: 7/15	ALL + RASB: 8/13 None: 8/15	0.662	0.71 (0.16, 3.23); p = 0.662
FEB + RASB vs. None	16	FEB + RASB: 1/16 None: 15/16	FEB + RASB: 0/1 None: 7/15	FEB + RASB: 1/1 None: 8/15	1.000	N/A
RASB vs. ALL	33	RASB: 17/33 ALL: 16/33	RASB: 14/17 ALL: 9/16	RASB: 3/17 ALL: 7/16	0.103	3.63 (0.74, 17.81); p = 0.112
ALL + RASB vs. ALL	29	ALL + RASB: 13/29 ALL: 16/29	ALL + RASB: 5/13 ALL: 9/16	ALL + RASB: 8/13 ALL: 7/16	0.340	0.49 (0.11, 2.16); p = 0.343
FEB + RASB vs. ALL	17	FEB + RASB: 1/17 ALL: 16/17	FEB + RASB: 0/1 ALL: 9/16	FEB + RASB: 1/1 ALL: 7/16	0.471	N/A
ALL + RASB vs. RASB	30	ALL + RASB: 13/30 RASB: 17/30	ALL + RASB: 5/13 RASB: 14/17	ALL + RASB: 8/13 RASB: 3/17	**0.023**	**0.13 (0.03, 0.71); p = 0.019** **[7.47 (1.40, 39.84); p = 0.019]**
FEB + RASB vs. RASB	18	FEB + RASB: 1/18 RASB: 17/18	FEB + RASB: 0/1 RASB: 14/17	FEB + RASB: 1/1 RASB: 3/17	0.222	N/A
FEB + RASB vs. ALL + RASB	14	FEB + RASB: 1/14 ALL + RASB: 13/14	FEB + RASB: 0/1 ALL + RASB: 5/13	FEB + RASB: 1/1 ALL + RASB: 8/13	1.000	N/A
Rehydration	56	Yes: 55/56 No: 1/56	Yes: 32/55 No: 1/1	Yes: 23/55 No: 0/1	1.000	N/A
Insulin	60	Yes: 8/60 No: 52/60	Yes: 6/8 No: 28/52	Yes: 2/8 No: 24/52	0.446	2.57 (0.47, 13.94); p = 0.274
Calcium gluconate	60	Yes: 4/60 No: 56/60	Yes: 4/4 No: 30/56	Yes: 0/4 No: 26/56	0.126	N/A
RRT	62	Yes: 23/62 No: 39/62	Yes: 12/23 No: 22/39	Yes: 11/23 No: 17/39	0.746	0.84 (0.30, 2.37); p = 0.746

Although the higher Cairo-Bishop clinical severity at STLS presentation was not associated with STLS-related death (*p* = 0.784), it was significantly associated with the need for RRT [*p* = 0.001; OR, 6.46; 95%CI, 2.08 to 20.08, *p*_*OR*_ = 0.001]. Patients receiving allopurinol were less likely to require RRT compared to those not receiving this medication [*p* = 0.021; OR, 0.29; 95%CI, 0.10 to 0.85, *p*_*OR*_ = 0.024] or those receiving rasburicase [*p* = 0.027; OR, 0.20; 95%CI, 0.05 to 0.87, *p*_*OR*_ = 0.032]. Notably, patients to which RRT was applied had significantly higher LDH levels at the STLS diagnosis than those not reaching RRT (*p* = 0.018, Table 4).

**Table 4:** Unified analyses on patients with solid tumors developing spontaneous tumor lysis syndrome (STLS). Characteristics of patients according to whether they needed renal replacement therapy (RRT) or not. Continuous data are presented as mean ± SD or median (1st quartile, 3rd quartile). All laboratory markers were measured at the point of the first establishment of the laboratory STLS. “Yes” refers to those exposed to the factor (1st column) whereas “No” to those not. Abbreviations: N, number of individuals whose data on the outcome was available; OR, odds ratio; CI, confidence interval; STLS, spontaneous tumor lysis syndrome; NA, not applicable; LDH, lactate dehydrogenase; WBC, white blood cells; ALL, allopurinol; FEB, febuxostat; RASB, rasburicase; RRT, renal replacement therapy

Factor	N	Overall	Need for RRT	No Need for RRT	p-value	Univariate logistic regression, Crude OR (95% CI); p-value
Age	66	59 (49, 69)	59 (49, 66)	60 (50, 69)	0.921	1.01 (0.97, 1.04); p = 0.727
Sex (female vs. male)	67	Female: 30/67 Male: 37/67	Female: 11/30 Male: 14/37	Female: 19/30 Male: 23/37	0.921	0.95 (0.35, 2.58); p = 0.921
Charlson Comorbidity Index	65	7 (6, 8)	7 (6, 8)	7 (6, 9)	0.445	0.85 (0.65, 1.12); p = 0.251
Diagnosis of the primary tumor along with STLS	65	Yes: 36/65 No: 29/65	Yes: 12/36 No: 12/29	Yes: 24/36 No: 17/29	0.504	0.71 (0.26, 1.95); p = 0.505
Time between diagnosis of the primary tumor and STLS (days)	54	4.5 (0, 30)	9.5 (2.5, 77)	1.5 (0, 12)	0.098	1.000 (0.997, 1.002); p = 0.928
Any treatment for primary tumor	64	Yes: 11/64 No: 53/64	Yes: 5/11 No: 18/53	Yes: 6/11 No: 35/53	0.505	1.62 (0.43, 6.04); p = 0.472
Surgical treatment of the primary site	65	Yes: 7/65 No: 58/65	Yes: 4/7 No: 19/58	Yes: 3/7 No: 39/58	0.233	2.74 (0.56, 13.48); p = 0.216
Stage	65	I: 1/65 II: 1/65 III: 5/65 IV: 58/65	I: 0/1 II: 0/1 III: 3/5 IV: 22/58	I: 1/1 II: 1/1 III: 2/5 IV: 36/58	0.767	(with III as a reference) IV: 0.41 (0.06, 2.63); p = 0.346
Tumor size	29	8.7 (6, 13)	8 (4, 14)	9.5 (6, 13)	0.770	0.99 (0.86, 1.14); p = 0.905
Lymph nodes	34	No: 1/34 Regional: 8/34 Distal: 25/34	No: 0/1 Regional: 5/8 Distal: 7/25	No: 1/1 Regional: 3/8 Distal: 18/25	0.145	(with “regional” as a reference) Distal: 0.23 (0.04, 1.25); p = 0.089
Metastasis	66	Yes: 58/66 No: 8/66	Yes: 22/58 No: 3/8	Yes: 36/58 No: 5/8	1.000	1.02 (0.22, 4.69); p = 0.981
Metastatic site(s)	65	Liver: 20/65 Lung: 5/65 Bones: 2/65 Liver & lungs: 12/65 Liver & bones: 9/65 Lung & bones: 1/65 Liver, lung & bones: 3/65 Metastatic but no liver, lung, or bones: 5/65 No metastasis: 8/65	Liver: 6/20 Lung: 2/5 Bones: 1/2 Liver & lungs: 6/12 Liver & bones: 2/9 Lung & bones: 0/1 Liver, lung & bones: 1/3 Metastatic but no liver, lung, or bones: 3/5 No metastasis: 3/8	Liver: 14/20 Lung: 3/5 Bones: 1/2 Liver & lungs: 6/12 Liver & bones: 7/9 Lung & bones: 1/1 Liver, lung & bones: 2/3 Metastatic but no liver, lung, or bones: 2/5 No metastasis: 5/8	0.871	(with “no metastasis” as a reference): Liver: 0.71 (0.13, 3.99); p = 0.702 Lung: 1.11 (0.11, 10.99); p = 0.928 Bones: 1.67 (0.07, 37.73); p = 0.748 Liver & Lung: 1.67 (0.27, 10.33); p = 0.583 Liver & Bones: 0.48 (0.06, 3.99); p = 0.494 Liver, lung & bones: 0.83 (0.05, 13.63); p = 0.898 Metastatic but no liver, lung, or bones: 2.50 (0.25, 24.72); p = 0.433
Liver (as one of the metastatic sites) vs. No metastasis at all	52	Liver: 44/52 No metastasis: 8/52	Liver: 15/44 No metastasis: 3/8	Liver: 29/44 No metastasis: 5/8	1.000	0.86 (0.18, 4.11); p = 0.852
Lung (as one of the metastatic sites) vs. No metastasis	29	Lung: 21/29 No metastasis: 8/29	Lung: 9/21 No metastasis: 3/8	Lung: 12/21 No metastasis: 5/8	1.000	1.25 (0.23, 6.65); p = 0.794
Bones (as one of the metastatic sites) vs. No metastasis	23	Bones: 15/23 No metastasis: 8/23	Bones: 4/15 No metastasis: 3/8	Bones: 11/15 No metastasis: 5/8	0.657	0.61 (0.10, 3.79); p = 0.592
Liver (as one of the metastatic sites) vs. No liver metastasis	64	Yes: 44/64 No: 20/64	Yes: 15/44 No: 8/20	Yes: 29/44 No: 12/20	0.648	0.78 (0.26, 2.31); p = 0.648
Lung (as one of the metastatic sites) vs. No lung metastasis	64	Yes: 21/64 No: 43/64	Yes: 9/21 No: 14/43	Yes: 12/21 No: 29/43	0.420	1.55 (0.53, 4.55); p = 0.421
Bones (as one of the metastatic sites) vs. No bone metastasis	64	Yes: 15/64 No: 49/64	Yes: 4/15 No: 19/49	Yes: 11/15 No: 30/49	0.392	0.57 (0.16, 2.07); p = 0.396
Time between STLS diagnosis and metastasis (days)	54	0 (0, 5)	0 (0, 6)	0 (0, 0)	0.312	1.002 (0.998, 1.006); p = 0.404
Treatment of metastasis	57	Yes: 1/57 No: 56/57	Yes: 1/1 No: 19/56	Yes: 0/1 No: 37/56	0.351	Ν/Α
Surgical treatment of metastasis	57	Yes: 1/57 No: 56/57	Yes: 1/1 No: 19/56	Yes: 0/1 No: 37/56	0.351	Ν/Α
Cairo Bishop clinical STLS severity on presentation	63	Mild or No Clinical STLS: 39/63 Severe Clinical STLS: 24/63	Mild or No Clinical STLS: 8/39 Severe Clinical STLS: 15/24	Mild or No Clinical STLS: 31/39 Severe Clinical STLS: 9/24	**0.001**	**6.46 (2.08, 20.08); p = 0.001**
Cairo Bishop clinical STLS severity on presentation	56	Mild Clinical STLS: 32/56 Severe Clinical STLS: 24/56	Mild Clinical STLS: 8/32 Severe Clinical STLS: 15/24	Mild Clinical STLS: 24/32 Severe Clinical STLS: 9/24	**0.005**	**5.00 (1.58, 15.80); p = 0.006**
Acute kidney injury	67	Yes: 55/67 No: 12/67	Yes: 23/55 No: 2/12	Yes: 32/55 No: 10/12	0.186	3.59 (0.72, 17.98); p = 0.119
Cardiac arrhythmia	67	Yes: 3/67 No: 64/67	Yes: 3/3 No: 22/64	Yes: 0/3 No: 42/64	**0.048**	N/A
New-onset seizure	67	Yes: 1/67 No: 66/67	Yes: 1/1 No: 24/66	Yes: 0/1 No: 42/66	0.373	N/A
Symptomatic hypocalcemia	67	Yes: 2/67 No: 65/67	Yes: 1/2 No: 24/65	Yes: 1/2 No: 41/65	1.000	1.71 (0.10, 28.58); p = 0.709
Blood urea nitrogen (mg/dl)	36	73.4 (51.5, 97)	79.1 (42, 100.3)	68.8 (54, 87.74)	0.638	1.00 (0.99, 1.02); p = 0.595
Serum creatinine (mg/dl)	61	3.4 (2.1, 4.6)	4.2 (3.4, 5.9)	2.4 (1.7, 4.0)	**0.001**	**1.43 (1.08, 1.89); p = 0.014**
Uric acid (mg/dl)	64	15.0 (13.0, 20.2)	17.4 (14.2, 22.4)	14.5 (11.6, 16.6)	**0.006**	**1.19 (1.05, 1.34); p = 0.005**
Potassium (mmol/l)	59	5.99 ± 0.93	6.04 ± 0.94	5.96 ± 0.93	0.769	1.09 (0.62, 1.93); p = 0.764
Phosphorus (mg/dl)	64	6.8 (5.1, 8.3)	7.0 (5.1, 8.9)	6.1 (5.1, 7.9)	0.276	1.07 (0.94, 1.22); p = 0.324
Calcium (mg/dl)	52	8.32 ± 1.50	8.08 ± 1.71	8.46 ± 1.36	0.375	0.84 (0.57, 1.23); p = 0.368
Sodium (mmol/l)	23	132 (126, 139)	133.5 (126, 144)	131 (128, 137.4)	0.756	1.03 (0.96, 1.10); p = 0.414
LDH (U/l)	46	1 357 (864, 3 922)	2 771 (1 192.5, 11 961.5)	1 037 (847, 2 304)	**0.018**	**1.0003 (1.00006, 1.00061); p = 0.016**
WBC (/µl)	22	18 375 (13 800, 30 300)	16 950 (12 300, 32 000)	19 800 (14 300, 21 000)	0.815	1.0000 (0.9999, 1.0001); p = 0.365
RASB vs. No RASB	63	Yes: 32/63 No: 31/63	Yes: 15/32 No: 9/31	Yes: 17/32 No: 22/31	0.145	2.16 (0.76, 6.11); p = 0.148
ALL vs. No ALL	64	Yes: 32/64 No: 32/64	Yes: 8/32 No: 17/32	Yes: 24/32 No: 15/32	**0.021**	**0.29 (0.10, 0.85); p = 0.024**
FEB vs. No FEB	63	Yes: 1/63 No: 62/63	Yes: 1/1 No: 23/62	Yes: 0/1 No: 39/62	0.381	N/A
At least 1 of ALL/FEB vs. No ALL/FEB	64	Yes: 33/64 No: 31/64	Yes: 9/33 No: 16/31	Yes: 24/33 No: 15/31	**0.046**	**0.35 (0.12, 0.99); p = 0.049**
At least 1 of ALL/FEB/RASB vs. No ALL/FEB/RASB	64	Yes: 50/64 No: 14/64	Yes: 19/50 No: 6/14	Yes: 31/50 No: 8/14	0.742	0.82 (0.25, 2.72); p = 0.742
ALL/FEB + RASB vs. No ALL/FEB + RASB	63	Yes: 15/63 No: 48/63	Yes: 5/15 No: 19/48	Yes: 10/15 No: 29/48	0.663	0.76 (0.23, 2.58); p = 0.664
ALL + RASB vs. No ALL + RASB	63	Yes: 14/63 No: 49/63	Yes: 4/14 No: 20/49	Yes: 10/14 No: 29/49	0.405	0.58 (0.16, 2.11); p = 0.409
FEB + RASB vs. No FEB + RASB	63	Yes: 1/63 No: 62/63	Yes: 1/1 No: 23/62	Yes: 0/1 No: 39/62	0.381	N/A
Urate-lowering treatment type	64	None: 14/64 ALL: 18/64 RASB: 17/64 ALL + RASB: 14/64 FEB + RASB: 1/64	None: 6/14 ALL: 4/18 RASB: 10/17 ALL + RASB: 4/14 FEB + RASB: 1/1	None: 8/14 ALL: 14/18 RASB: 7/17 ALL + RASB: 10/14 FEB + RASB: 0/1	0.109	See breakdown
ALL vs. None	32	ALL: 18/32 None: 14/32	ALL: 4/18 None: 6/14	ALL: 14/18 None: 8/14	0.267	0.38 (0.08, 1.77); p = 0.218
RASB vs. None	31	RASB: 17/31 None: 14/31	RASB: 10/17 None: 6/14	RASB: 7/17 None: 8/14	0.479	1.90 (0.45, 7.98); p = 0.378
ALL + RASB vs. None	28	ALL + RASB: 14/28 None: 14/28	ALL + RASB: 4/14 None: 6/14	ALL + RASB: 10/14 None: 8/14	0.430	0.53 (0.11, 2.56); p = 0.433
FEB + RASB vs. None	15	FEB + RASB: 1/15 None: 14/15	FEB + RASB: 1/1 None: 6/14	FEB + RASB: 0/1 None: 8/14	0.467	N/A
RASB vs. ALL	35	RASB: 17/35 ALL: 18/35	RASB: 10/17 ALL: 4/18	RASB: 7/17 ALL: 14/18	**0.027**	**5.00 (1.15, 21.80); p = 0.032** **[0.20 (0.05, 0.87); p = 0.032]**
ALL + RASB vs. ALL	32	ALL + RASB: 14/32 ALL: 18/32	ALL + RASB: 4/14 ALL: 4/18	ALL + RASB: 10/14 ALL: 14/18	0.703	1.40 (0.28, 6.98); p = 0.681
FEB + RASB vs. ALL	19	FEB + RASB: 1/19 ALL: 18/19	FEB + RASB: 1/1 ALL: 4/18	FEB + RASB: 0/1 ALL: 14/18	0.263	N/A
ALL + RASB vs. RASB	31	ALL + RASB: 14/31 RASB: 17/31	ALL + RASB: 4/14 RASB: 10/17	ALL + RASB: 10/14 RASB: 7/17	0.092	0.28 (0.06, 1.27); p = 0.098
FEB + RASB vs. RASB	18	FEB + RASB: 1/18 RASB: 17/18	FEB + RASB: 1/1 RASB: 10/17	FEB + RASB: 0/1 RASB: 7/17	1.000	N/A
FEB + RASB vs. ALL + RASB	15	FEB + RASB: 1/15 ALL + RASB: 14/15	FEB + RASB: 1/1 ALL + RASB: 4/14	FEB + RASB: 0/1 ALL + RASB: 10/14	0.333	N/A
Rehydration	58	Yes: 58/58 No: 0/58	Yes: 21/58	Yes: 37/58	N/A	N/A
Insulin	62	Yes: 7/62 No: 55/62	Yes: 5/7 No: 19/55	Yes: 2/7 No: 36/55	0.098	4.74 (0.84, 26.76); p = 0.078
Calcium gluconate	62	Yes: 3/62 No: 59/62	Yes: 0/3 No: 24/59	Yes: 3/3 No: 35/59	0.277	N/A

All comparisons of the examined variables with death and the need for RRT due to STLS as well as the results of the corresponding univariate logistic regression analyses are displayed in Tables 3 and 4. The sensitivity analysis that included cases with prior recent exposure to corticosteroids did not substantially change the findings (Tables S6, S7). The urate-lowering treatments per different metastatic sites are shown in Table S8.

## Discussion

To the best of our knowledge, this is the largest review systematically synthesizing available data on adult patients with solid tumors developing STLS. Another recent systematic review on TLS developing in patients with solid tumors identified only 32 cases of STLS [10], less than half of our included 66 cases. In accordance with overall data on TLS of solid tumors, our sample had a median age of about 60 years and predominantly consisted of males [6, 10]. Primary tumors were mostly large bulky masses (with a median largest transverse dimension of about 9 cm), commonly located in the lungs, colon, or liver [2, 6, 20, 46, 93]. Most patients had been diagnosed with metastatic disease mainly in the liver and/or lungs and had an unfavorable prognosis compared to previous studies on STLS of hematological malignancies [94]. There was a significantly higher likelihood of STLS-specific death in patients with metastatic disease – especially in those with liver or lungs involvement – compared to those without diagnosis of metastatic disease. A higher probability of death due to the syndrome was also observed in cases receiving rasburicase compared to those under no urate-lowering treatment or the allopurinol-rasburicase combination, while administration of allopurinol was associated with a significantly reduced likelihood of RRT need compared to no allopurinol or administration of rasburicase. Finally, CSTLS severity at the time of LTLS diagnosis was significantly associated with the need for RRT but not with death due to the syndrome.

Among factors related to high tumor burden, our data suggested that only metastatic disease, especially in the liver or lungs, significantly increases the likelihood of death due to STLS. Previous literature has proposed metastatic disease, especially in the liver, to be associated with an increased risk for TLS development [6, 20, 30, 32, 33, 36–38, 44, 58, 59, 93, 95, 96]. This is thought to occur due to increased tumor burden resulting in high purine pools and mechanical compression by the lesions [6, 32, 93, 95]. These may lead to hepatic synthetic dysfunction and impaired uric acid metabolism [6, 32, 93, 95]. High purine pools and mechanical compression of the non-cancerous tissue could also apply to cases of metastatic disease in the lungs. Similar mechanisms may be involved when examining the presence of metastatic disease not only as a risk factor for the spontaneous onset of the syndrome but also as an indicator of a worse prognosis. Interestingly, the liver and lungs were also among the three most reported primary sites of solid tumors resulting in STLS. Larger studies, leading to analyses with a higher power calculation, are required to detect differences in prognosis between different primary tumors and different metastatic sites. Regarding other markers of tumor burden, higher LDH levels on presentation were significantly associated with the need for RRT. As LDH may be, to some extent, indicative of cell lysis, elevated serum levels of the enzyme may reflect a more massive release of the nephrotoxic intracellular factors into the circulation [2, 8].

Interestingly, rasburicase was associated with increased rates of syndrome-related death (when compared to no urate-lowering treatment or combination with allopurinol) or requirement for RRT (when compared to allopurinol). A possible explanation of this observation could be that rasburicase was reserved for patients with a more severe course of STLS and was possibly applied at the point of clinical deterioration [43, 51, 69, 74, 92]. Rasburicase is well known for achieving a rapid and significant reduction in uric acid levels, both the newly produced and the preexisting ones [2, 97, 98]. In this context, its prophylactic administration is recommended in patients with hematological malignancies and at high risk for developing TLS [99]. On the contrary, allopurinol does not reduce preexisting uric acid levels but prevents further accumulation of this nephrotoxic metabolite [100]. Unfortunately, we were not able to further examine the hypothesis since the included case reports provided scarce quantitative data on the exact point at which rasburicase was administered. Considering that patients with metastatic disease in the liver or lungs were more likely to die than those without metastasis, we compared the administration of rasburicase between these groups (Table S8). Although only 1 out of 8 patients without metastasis received rasburicase (vs. 12 out of 43 or 8 out of 22 individuals with metastatic disease in the liver or lungs, respectively), no strong conclusions could be drawn from this small sample. Nevertheless, the effect of rasburicase on renal outcomes or mortality remains unclear and has mostly been studied in pediatric patients with hematological malignancies [97]. Data on adults mostly represent TLS on leukemias or lymphomas and has demonstrated that the use of rasburicase may be significantly associated with increased remission of the disease but not significantly associated with mortality at the first year [94]. The urate-lowering properties of allopurinol may partially explain its observed reduction in the need for RRT. Large-scale studies in adults with solid tumors and TLS are required to compare the safety of short- or long-term administration of urate-lowering agents.

Finally, our study indicated a significant association of Cairo-Bishop CSTLS severity at the time of its presentation with the requirement for RRT but not with death due to the syndrome. Considering that renal complications of STLS might be more frequent than manifestations such as uncontrollable seizures or life-threatening arrhythmias, the Cairo-Bishop grade of CSTLS at the time of presentation might have been predominantly determined by SeCr levels [12]. As more evidence on STLS of solid tumors is accumulated, future studies could investigate the prognostic value of updated classification systems that also account for factors related to tumor burden, such as metastatic disease. Of course, our data cannot question the importance of the Cairo-Bishop grading, since the analysis may have been underpowered and was based only on the status of patients at the point of LSTLS diagnosis. Besides, the classification system was designed more for distinguishing patients who require urgent medical interventions from those who do not [9, 12].

### Strengths & limitations

As of the time of writing, this is the largest review trying to synthesize all available data systematically and separately on adult patients with solid tumors developing STLS. We have attempted not only to describe the characteristics of these patients, or the parameters related to the syndrome but also to investigate which of these parameters may be associated with the need for RRT and STLS-caused death. The latter has not been addressed by previous studies in this subset of patients. Our findings may generate hypotheses in terms of prognostic factors of TLS in solid tumors and guide future clinical research.

However, our study also has certain limitations. Firstly, our small sample size has introduced a higher probability of type I error, leading to underpowered analyses, and not allowing to safely fit any multiple regression models. Secondly, the included population was highly heterogenous, consisting of various cancer histology, primary sites, and disease stages. Since a subgroup analysis by primary sites would be underpowered, we used the total sample investigating the effect on STLS-caused death and the need for RRT. Although we searched for all possible study designs, our findings were derived from case reports which are considered very low-quality evidence. Data from case reports are collected in a retrospective manner, and are, therefore, accompanied by a high risk for selection or confounding biases and subjectivity [13]. In addition, there is currently no specific cut-off timepoint that defines the spontaneous character of TLS. Despite using a cut-off of one month after the last administration of responsible agents, we found no studies that were excluded only due to this criterion. We should also highlight that Cairo-Bishop definitions of both LTLS and CTLS are based on cases of chemotherapy-induced TLS [12]; however, these are the most widely used definitions in the literature on TLS and the definitions used by the included studies. Of note, laboratory markers may have been measured at different timepoints during the evolution of STLS between the different case reports. The same issue applied to the Cairo-Bishop grade of CSTLS severity which may constantly change during hospitalization. We attempted to address these inconsistencies by recording clinical severity and laboratory measurements on patients’ presentation (i.e., at the point at which the diagnosis of LSTLS was established). Of course, this does not guarantee an identical time point of measurement. Reporting on dosing and timing of administration of urate-lowering agents and other supportive treatment was also inconsistent between the different case reports. Lastly, and due to the lack of control groups (i.e., patients not developing STLS), our data did not allow us to investigate for predisposing factors of STLS in a way other than narrative reporting of the triggering factors suggested by the authors. Larger studies that will better determine the parameters described above are required to compare different urate-lowering regimens, in terms of efficacy and safety, and identify currently less known triggering factors or predictors of worse prognosis. Nevertheless, conducting such studies is challenging when considering the rarity of TLS of solid tumors, especially when the analysis is restricted to cases with STLS.

## Conclusion

The synthesis of evidence on adult patients with solid tumors developing STLS demonstrated that metastatic disease, especially in the liver or lungs, and administration of rasburicase may be both associated with increased likelihood of death due to the syndrome compared to patients without any diagnosed metastasis and those receiving no urate-lowering treatment or the allopurinol-rasburicase combination, respectively. The need for RRT was found lower in patients receiving allopurinol than those not receiving allopurinol or those receiving rasburicase. The observed linkage between rasburicase and worse prognosis may be partially attributable to the potential selection of patients with a worse course of the syndrome to receive this specific agent. The CSTLS severity on its presentation was significantly associated with the need for RRT but not with death due to the syndrome. Based on all these findings, larger studies addressing the limitations of current literature and investigating the potential of underdiagnosis or underreporting of STLS in solid tumors are needed to compare the efficacy and safety of different management protocols and to identify unexplored triggering factors and predictors of worse prognosis.

## Electronic Supplementary Material

Below is the link to the electronic supplementary material.

Supplementary Material 1

## Data Availability

The datasets generated and analyzed during the current study are available from the corresponding author on reasonable request.

## References

[1] Coiffier B, Altman A, Pui CH, Younes A, Cairo MS (2008). Guidelines for the management of pediatric and adult tumor lysis syndrome: an evidence-based review. J Clin Oncol.

[2] Howard SC, Jones DP, Pui CH (2011). The tumor lysis syndrome. N Engl J Med.

[3] Howard SC, Trifilio S, Gregory TK, Baxter N, McBride A (2016). Tumor lysis syndrome in the era of novel and targeted agents in patients with hematologic malignancies: a systematic review. Ann Hematol.

[4] McBride A, Westervelt P (2012). Recognizing and managing the expanded risk of tumor lysis syndrome in hematologic and solid malignancies. J Hematol Oncol.

[5] Cairo MS, Coiffier B, Reiter A, Younes A (2010). Recommendations for the evaluation of risk and prophylaxis of tumour lysis syndrome (TLS) in adults and children with malignant diseases: an expert TLS panel consensus. Br J Haematol.

[6] Mirrakhimov AE, Ali AM, Khan M, Barbaryan A (2014). Tumor lysis syndrome in solid tumors: an up to date review of the literature. Rare Tumors.

[7] Noh GY, Choe DH, Kim CH, Lee JC (2008). Fatal tumor lysis syndrome during radiotherapy for non-small-cell lung cancer. J Clin Oncol.

[8] Alakel N, Middeke JM, Schetelig J, Bornhäuser M (2017). Prevention and treatment of tumor lysis syndrome, and the efficacy and role of rasburicase. Onco Targets Ther.

[9] Belay Y, Yirdaw K, Enawgaw B (2017). Tumor lysis syndrome in patients with hematological malignancies. J Oncol.

[10] Alqurashi RM, Tamim HH, Alsubhi ZD, Alzahrani AA, Tashkandi E (2022). Tumor lysis syndrome in patients with solid tumors: a systematic review of reported cases. Cureus.

[11] Tricco AC, Lillie E, Zarin W, O’Brien KK, Colquhoun H, Levac D (2018). PRISMA Extension for scoping reviews (PRISMA-ScR): Checklist and Explanation. Ann Intern Med.

[12] Cairo MS, Bishop M (2004). Tumour lysis syndrome: new therapeutic strategies and classification. Br J Haematol.

[13] Nissen T, Wynn R (2014). The clinical case report: a review of its merits and limitations. BMC Res Notes.

[14] Bramer WM, Giustini D, de Jonge GB, Holland L, Bekhuis T (2016). De-duplication of database search results for systematic reviews in EndNote. J Med Libr Assoc.

[15] Fay MP, Proschan MA (2010). Wilcoxon-Mann-Whitney or t-test? On assumptions for hypothesis tests and multiple interpretations of decision rules. Stat Surv.

[16] Kim HY (2017). Statistical notes for clinical researchers: Chi-squared test and Fisher’s exact test. Restor Dent Endod.

[17] Sperandei S (2014). Understanding logistic regression analysis. Biochem Med (Zagreb).

[18] Bujang MA, Sa’at N, Sidik T, Joo LC (2018). Sample size guidelines for logistic regression from Observational Studies with large Population: emphasis on the Accuracy between Statistics and Parameters based on Real Life Clinical Data. Malays J Med Sci.

[19] van Smeden M, Moons KGM, de Groot JAH, Collins GS, Altman DG, Eijkemans MJC (2018). Sample size for binary logistic prediction models: beyond events per variable criteria. Stat Methods Med Res.

[20] Caravaca-Fontán F, Martínez-Sáez O, Pampa-Saico S, Olmedo ME, Gomis A, Garrido P (2017). Tumor lysis syndrome in solid tumors: clinical characteristics and prognosis. Med Clin (Barc).

[21] Hsu HH, Chen YC, Tian YC, Chan YL, Kuo MC, Tang CC (2009). Role of serum sodium in assessing hospital mortality in cancer patients with spontaneous tumour lysis syndrome inducing acute uric acid nephropathy. Int J Clin Pract.

[22] Borne E, Serafi R, Piette F, Mortier L (2009). Tumour lysis syndrome induced by corticosteroid in metastatic melanoma presenting with initial hyperkalemia. J Eur Acad Dermatol Venereol.

[23] Habib GS, Saliba WR (2002). Tumor lysis syndrome after hydrocortisone treatment in metastatic melanoma: a case report and review of the literature. Am J Med Sci.

[24] Kalter JA, Allen J, Yang Y, Willing T, Evans E (2020). Spontaneous tumor lysis syndrome in an adenocarcinoma of unknown origin. Cureus.

[25] Lin CJ, Hsieh RK, Lim KH, Chen HH, Cheng YC, Wu CJ (2007). Fatal spontaneous tumor lysis syndrome in a patient with metastatic, androgen-independent prostate cancer. South Med J.

[26] McGhee-Jez A, Batra V, Sunder T, Rizk S (2018). Spontaneous tumor lysis syndrome as presenting sign of metastatic prostate Cancer. Cureus.

[27] Meeks MW, Hammami MB, Robbins KJ, Cheng KL, Lionberger JM (2016). Tumor lysis syndrome and metastatic melanoma. Med Oncol.

[28] Agarwala R, Batta A, Suryadevera V, Kumar V, Sharma V, Rana SS (2017). Spontaneous tumour lysis syndrome in hepatocellular carcinoma presenting with hypocalcemic tetany: an unusual case and systematic literature review. Clin Res Hepatol Gastroenterol.

[29] Alaigh V, Datta D (2017). Spontaneous tumor lysis syndrome due to Uterine Leiomyosarcoma with Lung Metastases. Case Rep Crit Care.

[30] Alan AM, Alan O (2020). A case of spontaneous tumor lysis syndrome in extensive-stage small-cell lung cancer: a rare oncologic emergency. Turk J Emerg Med.

[31] Ali AM, Barbaryan A, Zdunek T, Khan M, Voore P, Mirrakhimov AE (2014). Spontaneous tumor lysis syndrome in a patient with cholangiocarcinoma. J Gastrointest Oncol.

[32] Amiri FS (2015). Concurrent acute spontaneous tumor lysis syndrome complicated with multiple organ failure in a patient with pre-existing undiagnosed lung cancer. CEN Case Rep.

[33] Ammad Ud Din M, Hussain SA, Boppana LKT, Manogna D, Imran F (2020). Spontaneous tumor lysis syndrome in squamous cell carcinoma of the lung. Proc (Bayl Univ Med Cent).

[34] Berger R, Waler N, Schlumbrecht M, Huang M (2017). Spontaneous tumor lysis syndrome occurring in untreated uterine cancer. Gynecol Oncol Rep.

[35] Berringer R (2018). Spontaneous tumor lysis syndrome in a patient with newly diagnosed metastatic colonic adenocarcinoma. Cjem.

[36] Boonpheng B, Murtaza G, Ginn D (2017). Spontaneous tumor lysis syndrome in a patient with metastatic small cell Lung Cancer: a Case Report. Case Rep Oncol.

[37] Catania VE, Vecchio M, Malaguarnera M, Madeddu R, Malaguarnera G, Latteri S (2017). Tumor lysis syndrome in an extraskeletal osteosarcoma: a case report and review of the literature. J Med Case Rep.

[38] Chen KB, Xie WJ, Huang Y, Jin XL, Chen GF, Wu D (2019). Spontaneous tumor lysis syndrome in a patient with advanced gastric adenocarcinoma: a case report. Transl Cancer Res.

[39] Crittenden DR, Ackerman GL (1977). Hyperuricemic acute renal failure in disseminated carcinoma. Arch Intern Med.

[40] D’Alessandro V, Greco A, Clemente C, Sperandeo M, De Cata A, Di Micco C (2010). Severe spontaneous acute tumor lysis syndrome and hypoglycemia in patient with germ cell tumor. Tumori.

[41] Dean RK, Subedi R, Lee M (2018). Spontaneous tumor lysis syndrome in small cell lung cancer. Proc (Bayl Univ Med Cent).

[42] Dhakal P, Rai MP, Thrasher M, Sharma M. Spontaneous tumour lysis syndrome in small cell lung cancer: a rare phenomenon. BMJ Case Rep. 2018;2018. 10.1136/bcr-2018-224512 10.1136/bcr-2018-224512 PMC6011538 29898908

[43] Dong J, Cao T, Tanner N, Kundranda M (2020). When the Tumor Lyses: a Case Report on spontaneous tumor lysis syndrome. Case Rep Oncol.

[44] Durham CG, Herrington J, Seago S, Williams C, Holguin MH (2018). From skin to spontaneous lysis: a case of spontaneous tumor lysis syndrome in metastatic melanoma. J Oncol Pharm Pract.

[45] Feld J, Mehta H, Burkes RL (2000). Acute spontaneous tumor lysis syndrome in adenocarcinoma of the lung: a case report. Am J Clin Oncol.

[46] Gbaguidi X, Goodrich L, Roca F, Suel P, Chassagne P (2016). Bulky solid tumors in Elderly adults: beware of spontaneous tumor lysis syndrome. J Am Geriatr Soc.

[47] Goyal H, Sawhney H, Bekara S, Singla U (2014). Spontaneous acute tumour lysis syndrome in gastric adenocarcinoma: a case report and literature review. J Gastrointest Cancer.

[48] Goyal H, Sawhney H, Singh J (2012). Spontaneous fatal recurrent tumor lysis syndrome in ductal breast carcinoma. Community Oncol.

[49] Guardiani E, Chia SH (2011). Histiocytic sarcoma and tumor lysis syndrome: an unexpected Sequela of Neck mass. Laryngoscope.

[50] Hashem AAH, Dowod TAHM, Abdelmajeed MM (2010). Spontaneous tumor lysis syndrome: a case report and review of literature. Pak J Med Sci April-June.

[51] Ignaszewski M, Kohlitz P. Treatment-naïve spontaneous tumor lysis syndrome in metastatic prostate adenocarcinoma: An unusual suspect. Am J Emerg Med. 2017;35(9):1384.e1-.e2. 10.1016/j.ajem.2017.05.044 10.1016/j.ajem.2017.05.044 28587951

[52] Jallad B, Hamdi T, Latta S, Alhosaini MN, Kheir F, Iroegbu N (2011). Tumor lysis syndrome in small cell lung cancer: a case report and review of the literature. Onkologie.

[53] Kalmbach KE, Rahmat LT, Wos JA, Daniel NJ (2019). A rare oncologic emergency: spontaneous tumor lysis syndrome in metastatic Colon adenocarcinoma. Clin Pract Cases Emerg Med.

[54] Kanchustambham V, Saladi S, Patolia S, Stoeckel D. Spontaneous tumor lysis syndrome in small cell lung cancer. Cureus. 2017;9(2). 10.7759/cureus.1017 PMC5342890 28344911

[55] Kearney MR, Chen EY, Stenzel P, Corless CL, Deloughery TG, Zivney M (2019). Colorectal Cancer-Associated spontaneous tumor lysis syndrome: a Case Report and Review of the current literature. J Gastrointest Cancer.

[56] Kekre N, Djordjevic B, Touchie C (2012). Spontaneous tumour lysis syndrome. CMAJ.

[57] Kim YK, Ham JY, Lee WK, Song KE (2017). Spontaneous tumour lysis syndrome in cervical cancer. J Obstet Gynaecol.

[58] Martínez-Sáez O, Mezquita L, Caravaca-Fontan F, Reguera P, Molina J, Olmedo M (2016). Spontaneous tumor lysis syndrome in the setting of small cell lung cancer: report of two cases and review of the literature. Cancer Treat Res Commun.

[59] Mehrzad R, Saito H, Krahn Z, Feinstein A (2014). Spontaneous tumor lysis syndrome in a patient with metastatic hepatocellular carcinoma. Med Princ Pract.

[60] Mouallem M, Zemer-Wassercug N, Kugler E, Sahar N, Shapira-Frommer R, Schiby G (2013). Tumor lysis syndrome and malignant melanoma. Med Oncol.

[61] Myint PT, Butt HW, Alrifai T, Marin C (2019). Spontaneous tumor lysis syndrome secondary to small-cell neuroendocrine carcinoma of unknown origin: a Rare Case Report and Literature Review. Case Rep Oncol Med.

[62] Namdari N, Azarpira N (2019). Spontaneous tumor lysis syndrome in primitive neuroectodermal tumor. Middle East Journal of Cancer.

[63] Norberg SM, Oros M, Birkenbach M, Bilusic M (2014). Spontaneous tumor lysis syndrome in renal cell carcinoma: a case report. Clin Genitourin Cancer.

[64] Okamoto K, Kinoshita T, Shimizu M, Okura I, Kawada A, Mizobuchi K (2015). A case of spontaneous tumor lysis syndrome in a patient with ovarian Cancer. Case Rep Obstet Gynecol.

[65] Padhi P, Singh S (2012). Spontaneous tumor lysis syndrome in a patient with metastatic small cell carcinoma of the lung. J Cancer Sci Ther.

[66] Park SH, Lim JH, Jeong J, Lee SH, Cha HJ, Choi Y (2019). Bone marrow metastasis of small cell lung carcinoma with spontaneous tumor lysis syndrome without hepatic metastasis at diagnosis: first case report in Korea and review of literature. Blood Res.

[67] Parsi M, Rai M, Clay C (2019). You can’t always blame the chemo: a rare case of spontaneous tumor lysis syndrome in a patient with Invasive Ductal Cell Carcinoma of the breast. Cureus.

[68] Pentheroudakis G, O’Neill VJ, Vasey P, Kaye SB (2001). Spontaneous acute tumour lysis syndrome in patients with metastatic germ cell tumours. Report of two cases. Support Care Cancer.

[69] Pina Cabral J, Coelho J, Fortuna J, Rodrigues A (2021). Spontaneous tumor lysis syndrome in prostate Cancer. Cureus.

[70] Saini N, Pyo Lee K, Jha S, Patel S, Bonthu N, Kansagra A (2012). Hyperuricemic renal failure in nonhematologic solid tumors: a case report and review of the literature. Case Rep Med.

[71] Saleh RR, Rodrigues J, Lee TC. A tumour lysis syndrome in a chemotherapy naïve patient with metastatic pancreatic adenocarcinoma. BMJ Case Rep. 2015;2015. 10.1136/bcr-2014-207748 10.1136/bcr-2014-207748 PMC4322275 25634858

[72] Salmón-González Z, Vieitez-Santiago M, Martino-González M, Hernández JL, Alonso-Gutierrez J (2019). Spontaneous tumor lysis syndrome occurring in untreated gastric adenocarcinoma. QJM.

[73] Serling-Boyd N, Quandt Z, Allaudeen N (2017). Spontaneous tumor lysis syndrome in a patient with metastatic prostate cancer. Mol Clin Oncol.

[74] Shafie M, Teymouri A, Parsa S, Sadeghian A, Zarei Jalalabadi N (2022). Spontaneous tumor lysis syndrome in adrenal adenocarcinoma: a case report and review of the literature. J Med Case Rep.

[75] Shaforostova I, Fiedler R, Zander M, Pflumm J, März WJ (2020). Fatal spontaneous tumor lysis syndrome in a patient with metastatic Colon cancer: a clinical case of Rare Oncological Emergency. Case Rep Gastroenterol.

[76] Shenoy C (2009). Acute spontaneous tumor lysis syndrome in a patient with squamous cell carcinoma of the lung. QJM.

[77] Shukla DK, Gupta D, Aggarwal A, Kumar D (2017). A Case Report of newly diagnosed epithelial ovarian carcinoma presenting with spontaneous tumor lysis syndrome and its successful management with rasburicase. Indian J Med Paediatr Oncol.

[78] Sklarin NT, Markham M (1995). Spontaneous recurrent tumor lysis syndrome in breast cancer. Am J Clin Oncol.

[79] Sommerhalder D, Takalkar AM, Shackelford R, Peddi P (2017). Spontaneous tumor lysis syndrome in colon cancer: a case report and literature review. Clin Case Rep.

[80] Song M, Chan CCW, Stoeckel DA (2011). Spontaneous tumor lysis syndrome in metastatic melanoma. World J Oncol.

[81] Takeuchi N, Miyazawa S, Ohno Z, Yoshida S, Tsukamoto T, Fujiwara M (2016). A case of spontaneous tumor lysis syndrome in malignant melanoma. World J Oncol.

[82] Thapa J, Pandey S, Sitaula S, Dalal P, Varma D (2013). Spontaneous tumor lysis syndrome-a rare occurrence in endometrial/ovarian carcinoma. Crit Care Med.

[83] Tuharska Z, Galloway S, Stockman A, Damaskos D (2022). Spontaneous tumour lysis syndrome secondary to metastatic gallbladder adenocarcinoma: a Case Report and Reflection. Indian J Surg.

[84] Umar J, Kalakonda A, Panebianco L, Kaur G, John S (2017). Severe case of tumor lysis syndrome presenting spontaneously in a metastatic pancreatic adenocarcinoma patient. Pancreas.

[85] Vaisban E, Braester A, Mosenzon O, Kolin M, Horn Y (2003). Spontaneous tumor lysis syndrome in solid tumors: really a rare condition?. Am J Med Sci.

[86] Vieceli T, Tavares ALJ, de Moraes RP, Faulhaber GAM (2020). Metastatic adult neuroblastoma with spontaneous tumor lysis syndrome. Autops Case Rep.

[87] Wang Y, Yuan C, Liu X (2014). Cutaneous metastatic adenocarcinoma complicated by spontaneous tumor lysis syndrome: a case report. Oncol Lett.

[88] Watanabe S, Nanke I, Uchidate K, Machida T, Igarashi A, Kobashi K (2022). Case report of recurrent spontaneous tumor lysis syndrome in a patient with esophageal cancer recovered via chemotherapy. Int Cancer Conf J.

[89] Weerasinghe C, Zaarour M, Arnaout S, Garcia G, Dhar M (2015). Spontaneous tumor lysis syndrome in small-cell lung Cancer: a rare complication. World J Oncol.

[90] Woo IS, Kim JS, Park MJ, Lee MS, Cheon RW, Chang HM (2001). Spontaneous acute tumor lysis syndrome with advanced gastric cancer. J Korean Med Sci.

[91] Zakharia Y, Mansour J, Vasireddi S, Zakharia K, Fatakhov E, Koch C (2014). Tumor lysis syndrome in a retroperitoneal sarcoma. J Investig Med High Impact Case Rep.

[92] Chango Azanza JJ, Mathew Thomas V, Calle Sarmiento PM, Singh M, Alexander SA (2020). Spontaneous tumor lysis syndrome due to Endometrial Carcinoma. Cureus.

[93] Gemici C (2006). Tumour lysis syndrome in solid tumours. Clin Oncol (R Coll Radiol).

[94] Abdel-Nabey M, Chaba A, Serre J, Lengliné E, Azoulay E, Darmon M (2022). Tumor lysis syndrome, acute kidney injury and disease-free survival in critically ill patients requiring urgent chemotherapy. Ann Intensive Care.

[95] Barrett-Campbell O, Cook J, Brown J, Taiwo E, McFarlane SI (2019). Tumor lysis syndrome after hepatic artery embolization in a patient with neuroendocrine tumor of unknown primary. Am J Med Case Rep.

[96] Mirrakhimov AE, Voore P, Khan M, Ali AM (2015). Tumor lysis syndrome: a clinical review. World J Crit Care Med.

[97] Cheuk DK, Chiang AK, Chan GC, Ha SY (2017). Urate oxidase for the prevention and treatment of tumour lysis syndrome in children with cancer. Cochrane Database Syst Rev.

[98] Pession A, Masetti R, Gaidano G, Tosi P, Rosti G, Aglietta M (2011). Risk evaluation, prophylaxis, and treatment of tumor lysis syndrome: consensus of an italian expert panel. Adv Ther.

[99] Jones GL, Will A, Jackson GH, Webb NJ, Rule S (2015). Guidelines for the management of tumour lysis syndrome in adults and children with haematological malignancies on behalf of the British Committee for Standards in Haematology. Br J Haematol.

[100] Coiffier B, Mounier N, Bologna S, Fermé C, Tilly H, Sonet A (2003). Efficacy and safety of rasburicase (recombinant urate oxidase) for the Prevention and Treatment of Hyperuricemia during induction chemotherapy of aggressive Non-Hodgkin’s lymphoma: results of the GRAAL1 (Groupe d’Etude des lymphomes de l’Adulte trial on rasburicase activity in adult lymphoma) study. J Clin Oncol.

