# Supplementary material for: Spontaneous tumor lysis syndrome in patients with solid tumors: a scoping review of the literature
Source: Med Oncol. 2023 Jul 11;40(8):233. doi: 10.1007/s12032-023-02108-4 (PMC10335952; doi:10.1007/s12032-023-02108-4)
Supplement: Supplementary file 1 — Supplementary Material 1 [file 12032_2023_2108_MOESM1_ESM.docx]

**Title: Spontaneous tumor lysis syndrome in patients with solid tumors: a scoping review of the literature**

Authors: Michail Papapanou ^1,2^, Anastasios E. Athanasopoulos ^1*^, Eleni Georgiadi ^3,4*^, Stefanos A. Maragkos ^1*^, Michalis Liontos ^5^, Dimitrios C. Ziogas ^6^, Dimitrios Damaskos ^7^, Dimitrios Schizas ^8^

*Equal second contributors

^1^ Society of Junior Doctors, 15123 Athens, Greece

^2^ Second Department of Obstetrics and Gynecology, "Aretaieion Hospital", Medical School, National and Kapodistrian University of Athens, 76 Vas. Sofias Ave., 11528 Athens, Greece

^3^ 2nd Department of Radiology, Medical School, University General Hospital "Attikon", National and Kapodistrian University of Athens, 1 Rimini Str., 12462, Haidari/Athens, Greece

^4^ School of Medicine, National and Kapodistrian University of Athens, 11527 Athens, Greece

^5^ Department of Clinical Therapeutics, Division of Oncology, National and Kapodistrian University of Athens, Alexandra Hospital, 80 Vas. Sofias Ave., 10679 Athens, Greece

^6^ First Department of Internal Medicine, Unit of Medical Oncology, National and Kapodistrian University of Athens, Laikon General Hospital, 17 Agiou Thoma Str., 11527 Athens, Greece.

^7^ Department of Upper GI Surgery, Royal Infirmary of Edinburgh, Edinburgh, Scotland, UK

^8^ First Department of Surgery, National and Kapodistrian University of Athens, Laikon General Hospital, 17 Agiou Thoma Str., 11527 Athens, Greece

**Corresponding author.** Michail Papapanou, M.D., Second Department of Obstetrics and Gynecology, "Aretaieion Hospital", Medical School, National and Kapodistrian University of Athens, 76 Vas. Sofias Ave., 11528 Athens, Greece; email: mgpapapanou@gmail.com; **ORCID ID: 0000-0001-8331-2402**

**Supplementary Materials - Index**

| **Supplementary Methods** |  |
| --- | --- |
| Collected data items | *pag. 3* |
|  |  |
| **Supplementary Tables** |  |
| 1. Excluded studies with reasons | *pag. 5* |
| 2. Included studies and participants | *pag. 9* |
| 3. a. Characteristics of STLS after corticosteroids; b. Histological types and metastatic sites by primary tumor site. | *pag. 11* |
| 4. a. Characteristics of STLS regardless of prior recent exposure to corticosteroids; b. Histological types and metastatic sites by primary tumor site. | *pag. 14* |
| 5. Characteristics of included participants and STLS outcomes per site of the primary tumor. a. Patient demographics, tumor characteristics, and clinical characteristics and complications of STLS; b. Laboratory characteristics of STLS; c. Management of patients with STLS; d. Prognosis of patients with STLS. | *pag. 19* |
| 6. Unified analyses on patients with solid tumors developing spontaneous tumor lysis syndrome (STLS). Characteristics according to death or no death related to the STLS and its complications. | *pag. 26* |
| 7. Unified analyses on patients with solid tumors developing STLS. Characteristics of patients according to whether they needed renal replacement therapy (RRT) or not. | *pag. 28* |
| 8. Urate-lowering treatment per metastatic site of patients with solid tumors developing STLS: a. excluding, and b. including those with prior recent exposure to corticosteroids. | *pag. 30* |

**Supplementary Methods**

Collected data items.

*1. Participant and tumor-related characteristics*

- Age
- Sex
- First diagnosis of the primary tumor along with spontaneous tumor lysis syndrome (STLS): Yes/No
- Primary site tumor size (largest transverse dimension) at the time of STLS
- Histological description of malignancy (squamous/adenocarcinoma/sarcoma)
- Histological grading: low/intermediate/high
- Ki67
- Relevant tumor marker levels (e.g., CEA, CA19-9, CA125, AFP)
- Time between diagnosis of primary site cancer and TLS emergence
- Primary site
- Treatment for the primary tumor: yes/no
- Treatment for the primary tumor (describe): only surgical resection with negative margins, only surgical resection with positive microscopic margins, (neoadjuvant chemotherapy), and surgery and adjuvant chemotherapy, radiotherapy, etc.
- Time between the last administration of anti-cancer therapy and the emergence of TLS
- First diagnosis of metastatic site(s) along with STLS: yes/no
- Metastatic: yes/no
- Site of metastasis
- Time between diagnosis of metastasis and TLS emergence
- Histological description of metastatic site
- Stage (i.e., I-IV) at the time of TLS (as described by authors)
- Treatment for the metastatic tumor before STLS emergence: yes/no
- Treatment for the metastatic tumor (describe)
- Time of last administration of anti-cancer therapy for metastases

*2. TLS-related characteristics (clinical and laboratory presentation)*

- Potential triggers of the TLS (especially corticosteroids, biopsy)
- TLS-related symptoms (e.g., reduced level of consciousness, abdominal pain, etc.): describe
- Serum levels of blood urea nitrogen (BUN), creatinine (SeCr), uric acid, potassium, phosphorus (mg/dl), calcium (mg/dl), lactate dehydrogenase (LDH), pH, lactate, bicarbonate, sodium, white blood cell count (WBC)
- Description of other laboratory parameters
- Cairo/Bishop laboratory criteria fulfilled (conversions in mmol/L may be necessary for this step): Yes/No
- Laboratory TLS (yes/no) – to verify that the case stated as STLS is included as per Cairo-Bishop criteria for laboratory TLS.
- Cairo-Bishop grade of clinical severity on presentation
- Acute kidney injury (AKI): Yes/No
- Cardiac arrhythmia: Yes/No
- Seizure: Yes/No
- Symptomatic hypocalcemia: Yes/No
- Other complications: describe

*3. Management of STLS*

- Treatment for spontaneous TLS: describe
- Rasburicase: Yes/No
- If yes rasburicase: Dose
- Allopurinol: yes/no
- If yes allopurinol: dose
- Febuxostat: yes/no
- If yes febuxostat: dose
- At least one of allopurinol/febuxostat: yes/no
- At least one of allopurinol/febuxostat/rasburicase: yes/no
- Combination of allopurinol/febuxostat with rasburicase: yes/no
- Combination of allopurinol with rasburicase: yes/no
- Combination of febuxostat with rasburicase: yes/no
- Type of urate-lowering treatment
- Rehydration, calcium gluconate, insulin, b-agonist: yes/no and doses

*4. Prognosis of STLS*

- Need for renal replacement therapy due to STLS
- All-cause death: yes/no
- Death related to STLS or its direct complications
- Time from spontaneous TLS diagnosis to death
- Discharge: yes/no
- Time from spontaneous TLS diagnosis to discharge
- Recurrence: yes/no

**Supplementary Tables**

**Table S1.** Excluded studies with reasons. Abbreviation: SD, standard deviation.

| **Study identifier** | **Journal** | **Reason for exclusion** |
| --- | --- | --- |
| 1. Abdel-Nabey 2022   doi: 10.1186/s13613-022-00990-1 | Ann Intensive Care. | Hematological malignancy |
| 1. Abboud 2009   doi: 10.1159/000260525 | Case Rep Oncol. | Not spontaneous TLS |
| 1. Adler 2000   doi: 10.1097/00019048-200009090-00007 | Infectious Diseases in Clinical Practice | Hematological malignancy |
| 1. Alakel 2017   doi: 10.2147/OTT.S103864 | Onco Targets Ther. | Review |
| 1. Ansari 2020   doi: 10.7759/cureus.8257 | Cureus | Review |
| 1. Apiyo 2021   doi: 10.1177/00494755211029782 | Trop Doct. | Hematological malignancy |
| 1. Barrett-Campbell 2019   doi: 10.12691/ajmcr-7-7-4 | Am J Med Case Rep. | Not spontaneous TLS |
| 1. Bercovitz 2010   doi: 10.1097/MOP.0b013e32833499e5 | Curr Opin Pediatr. | Not spontaneous TLS |
| 1. Calvo Villas 2019   doi: 10.1016/j.medcli.2018.10.029 | Med Clin (Barc.) | Review |
| 1. Celkan 2013   doi: 10.4274/tpa.233 | Turk Arch Pediatr | Review |
| 1. Chanimov 2006 | Anesthesiology; 105:633–4 | Hematological malignancy |
| 1. Cheuk 2014   doi: 10.1002/14651858.CD006945.pub3 | Cochrane Database Syst Rev. | Review |
| 1. Coiffier 2007   doi: 10.1586/14737140.7.2.233 | Expert Rev Anticancer Ther. | Review |
| 1. Digumarti 2014   doi: 10.4103/0019-509X.138299 | Indian J Cancer. | Hematological malignancy |
| 1. Dhar 2016   doi: 10.4103/0259-1162.171446 | Anesth Essays Res.  . | Pediatric population |
| 1. Drakos 1994 | Am J Clin Oncol.  17 (6): 502-505 | Not spontaneous |
| 1. Esfahani 2015 | IJBC.  7(2): 97-99 | Pediatric population |
| 1. Farley-Hills 2001   doi: 10.1046/j.1460-9592.2001.00625.x | Paediatr Anaesth. | Hematological malignancy |
| 1. Frestad 2014   doi: 10.1136/bcr-2014-205002 | BMJ Case Rep. | Potential concurrent hematological malignancy |
| 1. Findakly 2020   doi: 10.7759/cureus.7395 | Cureus. | Review |
| 1. Firwana 2012   doi: 10.3810/pgm.2012.03.2540 | Postgrad Med. | Review |
| 1. Froilán Torres 2009   PMID: 19492906 | Rev Esp Enferm Dig. | Hematological malignancy |
| 1. Harada 2017   doi: 10.1155/2017/5103145 | Case Rep Oncol Med. | Concurrent hematological malignancy |
| 1. Hsieh 2009   doi: 10.3748/wjg.15.4726 | World J Gastroenterol. | Not spontaneous |
| 1. Hsu 2001   doi: 10.1081/jdi-100107369 | Ren Fail. | Hematological malignancy |
| 1. Hsu HH 2004 | Dialysis and Transplantation (Volume 33, Issue 6, Pages 316-325+347) | Population overlap/Duplicate |
| 1. Hsu HH 2004   PMID: 15151259 | J Nephrol. | Population overlap/Duplicate |
| 1. Hooman 2011   PMID: 21368393 | Iran J Kidney Dis. | Hematological malignancy |
| 1. Jaiswal 2015   doi: 10.1378/chest.2281864 | Chest | Not fulfilling criteria of laboratory STLS |
| 1. Jona 1999   doi: 10.1038/sj.jp.7200197 | J Perinatol. | Pediatric population |
| 1. Kalemkerian 1997 | Am J Med.  103:363–367 | Not spontaneous |
| 1. Kelkar 2021   doi: 10.7759/cureus.18108 | Cureus. | Review |
| 1. Kim 2015   doi: 10.3350/cmh.2015.21.1.85 | Clin Mol Hepatol. | Not spontaneous (presumably after transarterial chemoembolization) |
| 1. Kjellstrand 1974 | Arch Intern Med.  133: 349-359 | Hematological malignancy |
| 1. Lobe 1990 | J Ped Surg  25(2):249-250 | Pediatric population |
| 1. Micho 2018   doi: 10.1186/s12878-018-0117-0 | BMC Hematol. | Pediatric population |
| 1. Mika 2012   doi: 10.7314/apjcp.2012.13.8.3555 | Asian Pac J Cancer Prev. | Review |
| 1. Mirrakhimov 2015   doi: 10.5492/wjccm.v4.i2.130 | World J Crit Care Med. | Review |
| 1. Müller 2020   doi: 10.1024/1661-8157/a003527 | Praxis (Bern 1994) | Not spontaneous |
| 1. Murray 2011   doi: 10.3109/02688697.2011.566383 | Br J Neurosurg. | Pediatric population |
| 1. Muslimani 2011   PMID: 21618960 | Oncology (Williston Park). | Review |
| 1. Mughal 2010   doi: 10.1016/j.ctrv.2009.11.001 | Cancer Treat Rev. | Review |
| 1. Ñamendys-Silva 2015   doi: 10.2147/OAEM.S73684 | Open Access Emerg Med | Review |
| 1. Pession 2011   doi: 10.1007/s12325-011-0041-1 | Adv Ther. | Review |
| 1. Ponmudi 2018   doi: 10.1136/bcr-2017-223107 | BMJ Case Rep. | Pediatric population |
| 1. Prem 2008 | Biomedicine (Volume 28, Issue 2, Pages 69-72) | Review |
| 1. Rodriguez 2011   doi: 10.1358/dot.2011.47.8.1622067 | Drugs Today (Barc). | Review |
| 1. Rovelo-Lima 2010 | Gaceta Mexicana de Oncologia (Volume 9, Issue 4, Pages 175-179) | Review |
| 1. Roy-Chaudhury 2006   doi: 10.1038/sj.ki.5001696 | Kidney Int. | Hematological malignancy |
| 1. Russell 2020   doi: 10.1542/pir.2018-0243 | Pediatr Rev. | Review (Book Chapter) |
| 1. Sarno 2013   PMID: 25031988 | J Adv Pract Oncol. | Review |
| 1. Shenoy 2015   doi: 10.1007/s12291-014-0472-y | Indian J Clin Biochem. | Hematological malignancy |
| 1. Sobota 2014   PMID: 25141580 | Przegl Lek. | Review |
| 1. Turtureanu-Hanganu 2002   PMID: 14974215 | Rev Med Chir Soc Med Nat Iasi. | Review |

**Table S2.** Main characteristics of included studies and participants.

| **First author** | **Publication year** | **Study design** | **Country** | **Age** | **Sex** | **Exposure to corticosteroids** |
| --- | --- | --- | --- | --- | --- | --- |
| Agarwala | 2016 | Case report | India | 26 | Female | No |
| Alaigh | 2016 | Case report | USA | 58 | Female | No |
| Alan | 2020 | Case report | Turkey | 59 | Male | No |
| Ali | 2014 | Case report | USA | 66 | Male | No |
| Amiri | 2015 | Case report | Iran | 79 | Male | No |
| Ammad Ud Din | 2020 | Case report | USA | 57 | Female | No |
| Berger 1 | 2017 | Case report | USA | 65 | Female | No |
| Berger 2 | 2017 | Case report | USA | 33 | Female | No |
| Berringer | 2017 | Case report | Canada | 48 | Male | No |
| Boonpheng | 2017 | Case report | USA | 55 | Female | No |
| Borne | 2009 | Case report | France | 42 | Male | Yes |
| Caravaca-Fontán | 2017 | Retrospective cohort (only 9 patients eligible) | Spain | mean ± SD:  63 ± 13 | Male: 8 (88.9%) | No |
| Catania | 2017 | Case report | Italy | 65 | Female | No |
| Chango Azanza | 2020 | Case report | USA | 59 | Female | No |
| Chen | 2019 | Case report | China | 62 | Male | No |
| Crittenden | 1977 | Case report | USA | 50 | Male | No |
| D’Alessandro | 2010 | Case report | Italy | 22 | Male | No |
| Dean | 2018 | Case report | USA | 76 | Female | No |
| Dhakal | 2018 | Case report | USA | NR | Male | No |
| Dong | 2020 | Case report | USA | 59 | Male | No |
| Durham | 2017 | Case report | USA | 59 | Male | No |
| Feld | 2000 | Case report | Canada | 72 | Male | No |
| Gbaguidi | 2016 | Case report | France | 88 | Female | No |
| Goyal | 2014 | Case report | USA | 51 | Male | No |
| Goyal | 2012 | Case report | USA | 51 | Female | No |
| Guardiani | 2011 | Case report | USA | 27 | Female | No |
| Habib | 2002 | Case report | Israel | 56 | Female | Yes |
| Hashem | 2010 | Case report | Kuwait | 73 | Male | No |
| Hsu | 2009 | Retrospective cohort (only 1 case eligible) | Taiwan | 85 | Male | No |
| Ignaszewski | 2017 | Case report | USA | 69 | Male | No |
| Jallad | 2011 | Case report | USA | 75 | Female | No |
| Kalmbach | 2019 | Case report | USA | 66 | Female | No |
| Kalter | 2020 | Case report | USA | 77 | Female | Yes |
| Kanchustambham | 2017 | Case report | USA | 53 | Male | No |
| Kearney | 2018 | Case report | USA | 47 | Female | No |
| Kekre | 2012 | Case report | Canada | 76 | Male | No |
| Kim | 2017 | Case report | Korea | 35 | Female | No |
| Lin | 2007 | Case report | Taiwan | 72 | Male | Yes |
| Martínez-Sáez 1 | 2016 | Case report | Spain | 60 | Male | No |
| Martínez-Sáez 2 | 2016 | Case report | Spain | 64 | Male | No |
| McGhee-Jez | 2018 | Case report | USA | 49 | Male | Yes |
| Meeks | 2016 | Case report | USA | 46 | Male | Yes |
| Mehrzad | 2014 | Case report | USA | 70 | Male | No |
| Mouallem | 2013 | Case report | Israel | 69 | Male | No |
| Myint | 2019 | Case report | USA | 66 | Female | No |
| Namdari | 2019 | Case report | Iran | 22 | Male | No |
| Norberg | 2014 | Case report | USA | 56 | Male | No |
| Okamoto | 2015 | Case report | Japan | 62 | Female | No |
| Padhi | 2012 | Case report | USA | 73 | Female | No |
| Park | 2019 | Case report | Korea | 71 | Male | No |
| Parsi | 2019 | Case report | USA | 36 | Female | No |
| Pentheroudakis 1 | 2021 | Case report | UK | 24 | Male | No |
| Pentheroudakis 2 | 2021 | Case report | UK | 52 | Male | No |
| Pina Cabral | 2021 | Case report | Portugal | 58 | Male | No |
| Saini | 2012 | Case report | USA | 59 | Female | No |
| Saleh | 2015 | Case report | Canada | 56 | Female | No |
| Salmon-Gonzalez | 2019 | Case report | Spain | 79 | Male | No |
| Serling-Boyd | 2017 | Case report | USA | 56 | Male | No |
| Shafie | 2022 | Case report | Iran | 64 | Male | No |
| Shaforostova | 2020 | Case report | Germany | 47 | Female | No |
| Shenoy | 2009 | Case report | USA | 74 | Male | No |
| Shukla | 2017 | Case report | India | 49 | Female | No |
| Sklarin | 1995 | Case report | USA | 62 | Female | No |
| Sommerhalder | 2017 | Case report | USA | 49 | Female | No |
| Song | 2011 | Case report | USA | 46 | Male | No |
| Takeuchi | 2016 | Case report | Japan | 62 | Male | No |
| Thapa | 2013 | Case report | USA | 45 | Female | No |
| Tuharska | 2021 | Case report | UK | 77 | Female | No |
| Umar | 2017 | Case report | USA | 68 | Female | No |
| Vaisban 1 | 2005 | Case report | Israel | 72 | Male | No |
| Vaisban 2 | 2005 | Case report | Israel | 80 | Male | No |
| Vaisban 3 | 2005 | Case report | Israel | 82 | Female | No |
| Vieceli | 2020 | Case report | Brazil | 27 | Female | No |
| Wang | 2014 | Case report | China | 71 | Female | No |
| Watanabe | 2022 | Case report | Japan | 61 | Male | No |
| Weerasinghe | 2015 | Case report | USA | 65 | Male | No |
| Woo | 2001 | Case report | Korea | 36 | Male | No |
| Zakharia | 2014 | Case report | USA | 49 | Female | No |

**Table S3a.** Characteristics of included participants who developed tumor lysis syndrome after prior recent exposure to corticosteroids. Unless otherwise stated, categorical variables are presented as frequencies (%) and continuous variables are presented as median (first quartile, third quartile). Abbreviations: N, number of individuals whose data on the outcome was available; Q1, 1st quartile; Q3, 3rd quartile; SD, standard deviation; STLS, spontaneous tumor lysis syndrome; NA, not applicable; CSTLS, clinical spontaneous tumor lysis syndrome; LSTLS, laboratory spontaneous tumor lysis syndrome.

| **Variable** | **Case reports** | |
| --- | --- | --- |
|  | **N** | **Values** |
| Total N | 6 | |
| Age [median (Q1, Q3) / mean (SD)] | 6 | 52.5 (46.0, 72.0) |
| Sex, male (%) | 6 | 4 (66.7%) |
| Charlson Comorbidity Index on admission | 6 | 6.5 (6, 9) |
| Classified Charlson Comorbidity Index on admission | 6 | Severe: 6 (100%) |
| **Tumor characteristics** | | |
| Diagnosis of TLS along with the first diagnosis of the primary tumor | 6 | 2 (33.3%) |
| Time between diagnosis of the primary tumor and TLS (days) | 6 | 161 (3, 365) |
| Primary sites | 6 | Skin: 3 (50%) |
|  |  | Prostate: 2 (33.3%) |
|  |  | Unknown: 1 (16.7%) |
| Tumor size (largest dimension in cm) | 0 | N/A |
| Histological grade | 0 | N/A |
| Lymph nodes | 2 | Distal: 2 (100%) |
| Metastasis | 6 | 6 (100%) |
| Metastatic sites | 6 | Bones: 1 (16.67%) |
|  |  | Liver & lung: 1 (16.67%) |
|  |  | Liver & bones: 3 (50%) |
|  |  | Liver, lung & bones: 1 (16.67%) |
| Stage | 6 | IV: 6 (100%) |
| Had received any kind of treatment for the primary tumor before the TLS diagnosis | 6 | 3 (50.0%) |
| Had received surgical treatment for primary tumor before TLS diagnosis | 6 | 1 (16.7%) |
| Had received any kind of treatment for metastatic tumor before the TLS diagnosis | 5 | 1 (20%) |
| Had received surgical treatment for metastatic tumor before TLS diagnosis | 5 | 0 (0%) |
| Time between diagnosis of metastatic tumor(s) and TLS (days) | 6 | 11 (0, 180) |
| **TLS characteristics** | | |
| Acute kidney injury | 6 | 6 (100%) |
| Cardiac arrhythmia | 6 | 1 (16.7%) |
| New-onset seizure | 6 | 0 (0%) |
| Symptomatic hypocalcemia | 6 | 0 (0%) |
| Blood urea nitrogen (mg/dl) | 3 | 96 (49, 154) |
| Serum creatinine (mg/dl) | 5 | 5.4 (3.0, 6.1) |
| Uric acid (mg/dl) | 5 | 14.6 (12.2, 15.6) |
| Potassium (mmol/l) | 4 | 5.1 (4.3, 6.5) |
| Phosphorus (mg/dl) | 4 | 9.7 (8.7, 12.4) |
| Calcium (mg/dl) | 3 | 8.0 (6.6, 10.8) |
| Sodium (mmol/l) | 2 | 136 (133, 139) |
| Lactate dehydrogenase (U/l) | 3 | 4445 (1288, 20870) |
| White blood cells (/μL) | 0 | N/A |
| Cairo-Bishop clinical grade on admission | 5 | 2: 2 (40%) |
|  |  | 3: 3 (60%) |
| Cairo-Bishop clinical grade on admission (mild vs. severe) | 5 | Mild: 2 (40%) |
|  |  | Severe: 3 (60%) |
| **Management of TLS** | | |
| Received allopurinol | 6 | 3 (50%) |
| Received febuxostat | 6 | 0 (0%) |
| Received allopurinol/febuxostat | 6 | 3 (50%) |
| Received rasburicase | 6 | 3 (50%) |
| Received allopurinol/febuxostat/rasburicase | 6 | 6 (100%) |
| Received allopurinol/febuxostat and rasburicase | 6 | 0 (0%) |
| Received allopurinol and rasburicase | 6 | 0 (0%) |
| Received allopurinol and febuxostat | 6 | 0 (0%) |
| Received febuxostat and rasburicase | 6 | 0 (0%) |
| Type of urate-lowering treatment | 6 | Allopurinol monotherapy: 3 (50%) |
|  |  | Rasburicase monotherapy: 3 (50%) |
| Received insulin | 6 | 1 (16.7%) |
| Received calcium gluconate | 6 | 0 (0%) |
| Had a need for/received renal replacement therapy | 6 | 4 (66.7%) |
| **Prognosis of TLS** | | |
| Death related to TLS | 6 | 4 (66.7%) |
| All-cause death | 6 | 5 (83.3%) |
| Time between diagnosis of TLS and death (days) | 4 | 4.0 (2.5, 9.0) |
| Discharge | 3 | 2 (66.7%) |
| Time between diagnosis of TLS and discharge (days) | 1 | 12 |
| Recurrence | 6 | 0 (0%) |

**Table S3****b.** Histological types and metastatic sites by primary tumor site. Only data on patients who developed tumor lysis syndrome after prior recent exposure to corticosteroids are presented.

| **Primary tumor site** | **Histological types per primary tumor site** | **Metastatic sites per primary tumor site** |
| --- | --- | --- |
| Skin: 3 (50%) | Melanoma: 3 | Liver & bones: 2  Liver, lung & bones: 1 |
| Prostate: 2 (33.3%) | Adenocarcinoma: 2 | Bones: 1  Liver & bones: 1 |
| Unknown: 1 (16.7%) | Adenocarcinoma: 1 | Liver & lung: 1 |

**Table S4a.** Characteristics of included participants. Data on all patients (including both those with and without prior recent exposure to corticosteroids) are presented. Data on patients of the case reports and those of the cohort are separately presented. Unless otherwise stated, categorical variables are presented as frequencies (%) and continuous variables are presented as median (first quartile, third quartile). Abbreviations: N, number of individuals whose data on the outcome was available; Q1, 1st quartile; Q3, 3rd quartile; SD, standard deviation; TLS, tumor lysis syndrome; N/A, not applicable.

| **Variable** | **Case reports** | | **Cohort** |
| --- | --- | --- | --- |
|  | **N** | **Values** |  |
| Total N | 77 | | 9 |
| Age [median (Q1, Q3) / mean (SD)] | 76 | 59.5 (49.0, 70.5) | 63 (13) |
| Sex, male (%) | 77 | 42 (54.6%) | 8 (88.9%) |
| Charlson Comorbidity Index on admission | 75 | 7 (6, 9) | 7 (4, 9) |
| Classified Charlson Comorbidity Index on admission | 75 | Moderate: 3 (4%) | NA |
|  |  | Severe: 72 (96%) |  |
| **Tumor characteristics** | | | |
| Diagnosis of TLS along with the first diagnosis of the primary tumor | 75 | 42 (56%) | NA |
| Time between diagnosis of the primary tumor and STLS (days) | 64 | 5.5 (0, 47.5) | 37 (12, 49) |
| Primary sites | 77 | Lung: 15 (19.5%) | Lung: 3 (33.3%) |
|  |  | Skin: 6 (7.8%) | Stomach: 1 (11.1%) |
|  |  | Colon: 6 (7.8%) | Colon: 1 (11.1%) |
|  |  | Liver: 5 (6.5%) | NA |
|  |  | Prostate: 5 (6.5%) | NA |
|  |  | Uterus (Endometrium/Myometrium): 4 (5.2%) | Uterus (Endometrium): 1 (11.1%) |
|  |  | Stomach: 4 (5.2%) | NA |
|  |  | Breast: 3 (3.9%) | NA |
|  |  | Kidney: 3 (3.9%) | NA |
|  |  | Ovary: 2 (2.6%) | NA |
|  |  | Pancreas: 2 (2.6%) | NA |
|  |  | Adrenal gland: 2 (2.6%) | NA |
|  |  | Uterus – cervix: 1 (1.3%) | NA |
|  |  | Gallbladder: 1 (1.3%) | NA |
|  |  | Esophagus: 1 (1.3%) | Esophagus: 1 (11.1%) |
|  |  | Other: 10 (12.9%) | Other: 1 (11.1%) |
|  |  | Unknown: 7 (9.1%) | Unknown: 1 (11.1%) |
| Tumor size (largest dimension in cm) | 30 | 8.9 (6, 13) | NA |
| Histological grade | 26 | Low: 8 (30.8%) | NA |
|  |  | Intermediate: 2 (7.7%) |  |
|  |  | High: 16 (61.5%) |  |
| Lymph nodes | 37 | No: 1 (2.7%) | NA |
|  |  | Regional: 8 (21.6%) |  |
|  |  | Distal: 28 (75.7%) |  |
| Metastasis | 76 | 67 (88.2%) | NA |
| Metastatic sites | 67 | Liver: 21 (31.3%) | NA |
|  |  | Lung: 6 (9.0%) |  |
|  |  | Bones: 3 (4.5%) |  |
|  |  | Liver & lung: 13 (19.4%) |  |
|  |  | Liver & bones: 13 (19.4%) |  |
|  |  | Lung & bones: 1 (1.5%) |  |
|  |  | Liver, lung & bones: 4 (6.0%) |  |
|  |  | No liver, lung or bones (but other metastatic sites): 5 (7.4%) |  |
|  |  | Unknown (disease presented as metastatic but sites not reported): 1 (1.5%) |  |
| Stage | 74 | I: 1 (1.4%) | NA |
|  |  | II: 1 (1.4%) |  |
|  |  | III: 5 (6.8%) |  |
|  |  | IV: 67 (90.4%) |  |
| Had received any kind of treatment for primary tumor before STLS diagnosis | 75 | 15 (20.0%) | NA |
| Had received surgical treatment for primary tumor before STLS diagnosis | 75 | 9 (12.0%) | NA |
| Had received any kind of treatment for metastatic tumor before STLS diagnosis | 65 | 2 (3.1%) | NA |
| Had received surgical treatment for metastatic tumor before STLS diagnosis | 65 | 1 (1.5%) | NA |
| Time between diagnosis of metastatic tumor(s) and STLS (days) | 63 | 0 (0, 6) | NA |
| **STLS characteristics** | | | |
| Acute kidney injury | 77 | 65 (84.4%) | 9 (100%) |
| Cardiac arrhythmia | 77 | 4 (5.2%) | 3 (33.3%) |
| New-onset seizure | 77 | 1 (1.3%) | 5 (55.6%) |
| Symptomatic hypocalcemia | 77 | 2 (2.6%) | 1 (11.1%) |
| Blood urea nitrogen (mg/dl) | 42 | 73.9 (54, 100) | NA |
| Serum creatinine (mg/dl) [median (Q1, Q3) / mean (SD)] | 69 | 3.5 (2.1, 4.9) | 2.9 (1.5) |
| Uric acid (mg/dl) [median (Q1, Q3) / mean (SD)] | 72 | 15 (12.8, 20.3) | 16.3 (5.0) |
| Potassium (mmol/l) [mean (SD)] | 66 | 6.0 (1.0) | 6.2 (1.0) |
| Phosphorus (mg/dl) [median (Q1, Q3) / mean (SD)] | 71 | 6.9 (5.2, 8.8) | 7.3 (3.3) |
| Calcium (mg/dl) [mean (SD)] | 58 | 8.2 (1.6) | 8.3 (0.8) |
| Sodium (mmol/l) | 27 | 132 (126, 139) | NA |
| Lactate dehydrogenase (U/l) [median (Q1, Q3) / mean (SD)] | 50 | 1459.5 (899, 4023) | 1554 (1010) |
| White blood cells (/μL) | 23 | 16950 (12300, 30300) | NA |
| Cairo-Bishop clinical grade on admission | 71 | 0 (no CTLS): 7 (9.9%) |  |
|  |  | 1: 12 (16.9%) | NA |
|  |  | 2: 22 (31.0%) |  |
|  |  | 3: 26 (36.6%) |  |
|  |  | 4: 4 (5.6%) |  |
| Cairo-Bishop clinical grade on admission (no or mild vs. severe) | 71 | No or Mild CTLS: 41 (57.8%) | NA |
|  |  | Severe CTLS: 30 (42.2%) |  |
| Cairo-Bishop clinical grade on admission (mild vs. severe) | 64 | Mild CTLS: 34 (53.1%) | NA |
|  |  | Severe CTLS: 30 (46.9%) |  |
| **Management of STLS** | | | |
| Received allopurinol | 72 | 35 (48.6%) | NA |
| Received febuxostat | 71 | 1 (1.4%) | NA |
| Received allopurinol/febuxostat | 72 | 36 (50.0%) | NA |
| Received rasburicase | 71 | 36 (50.7%) | NA |
| Received allopurinol/febuxostat/rasburicase | 72 | 57 (79.2%) | NA |
| Received allopurinol/febuxostat and rasburicase | 71 | 15 (21.1%) | NA |
| Received allopurinol and rasburicase | 71 | 14 (19.7%) | NA |
| Received allopurinol and febuxostat | 71 | 0 (0%) | NA |
| Received febuxostat and rasburicase | 71 | 1 (1.4%) | NA |
| Type of urate-lowering treatment | 72 | Allopurinol monotherapy: 21 (29.2%) | NA |
|  |  | Febuxostat monotherapy: 0 (0%) |  |
|  |  | Rasburicase monotherapy: 21 (29.2%) |  |
|  |  | Allopurinol & Rasburicase: 14 (19.4%) |  |
|  |  | Febuxostat & Rasburicase: 1 (1.4%) |  |
|  |  | No urate-lowering treatment: 15 (20.8%) |  |
| Received insulin | 70 | 9 (12.9%) | NA |
| Received calcium gluconate | 70 | 4 (5.7%) | NA |
| Had a need for/received renal replacement therapy | 73 | 29 (39.7%) | 0 (0%) |
| **Prognosis of STLS** | | | |
| Death related to STLS | 71 | 40 (56.3%) | 7 (77.8%) |
| All-cause death | 71 | 57 (80.3%) | NA |
| Time between diagnosis of STLS and death (days) | 42 | 4 (1, 10) | NA |
| Discharge | 55 | 20 (36.4%) | NA |
| Time between diagnosis of STLS and discharge (days) | 10 | 11.5 (10, 14) | NA |
| Recurrence | 76 | 2 (2.6%) | NA |

**Table S4****b.** Histological types and metastatic sites by primary tumor site. Data on all patients (including both those with and without prior recent exposure to corticosteroids) are presented. Data on patients of the case reports and those of the cohort are separately presented.

| **Study type, N** | **Primary tumor site** | **Histological types per primary tumor site** | **Metastatic sites per primary tumor site** |
| --- | --- | --- | --- |
| Case reports, N = 77 | Lung: 15 (19.5%) | Squamous carcinoma: 5  Adenocarcinoma: 1  Small cell lung cancer: 8  Unknown: 1 | Liver: 9  Bones: 1  Liver & lungs: 1  Liver & bones: 1  Unknown: 1  No metastasis: 2 |
|  | Skin: 6 (7.8%) | Melanoma: 6 | Liver & lungs: 1  Liver & bones: 2  Liver, lung & bones: 2  Other: 1 |
|  | Colon: 6 (7.8%) | Adenocarcinoma: 6 | Liver: 3  Lung: 1  Liver & lung: 1  Liver & bones: 1 |
|  | Liver: 5 (6.5%) | Hepatocellular carcinoma: 3  Adenocarcinoma: 1  Unknown: 1 | Lung: 3  No metastasis: 1  Unknown: 1 |
|  | Prostate: 5 (6.5%) | Adenocarcinoma: 5 | Bones: 2  Liver & bones: 3 |
|  | Uterus (Endometrium/Myometrium): 4 (5.2%) | Endometrial adenocarcinoma: 2  Leiomyosarcoma: 1  Neuroendocrine carcinoma: 1 | Liver: 1  Lung: 1  No metastasis: 2 |
|  | Stomach: 4 (5.2%) | Adenocarcinoma: 4 | Liver: 2  Liver & bones: 1  Other: 1 |
|  | Breast: 3 (3.9%) | Adenocarcinoma: 3 | Liver & lung: 1  Liver, lung & bones: 1  No metastasis: 1 |
|  | Kidney: 3 (3.9%) | Adenocarcinoma: 1  Sarcoma: 1  Urothelial carcinoma: 1 | Liver & bones: 2  No metastasis: 1 |
|  | Ovary: 2 (2.6%) | Adenocarcinoma: 2 | Other: 1  No metastasis: 1 |
|  | Pancreas: 2 (2.6%) | Adenocarcinoma: 2 | Liver: 1  Liver & lung: 1 |
|  | Adrenal gland: 2 (2.6%) | Adenocarcinoma: 1  Pheochromocytoma: 1 | Liver: 1  No metastasis: 1 |
|  | Uterus – cervix: 1 (1.3%) | Squamous carcinoma: 1 | Lung & bones: 1 |
|  | Gallbladder: 1 (1.3%) | Adenocarcinoma: 1 | Liver: 1 |
|  | Esophagus: 1 (1.3%) | Squamous carcinoma: 1 | Liver & bones: 1 |
|  | Other: 10 (12.9%) | Melanoma of the eyeball: 1 | Liver: 1 |
|  |  | Retroperitoneal: 1 adenocarcinoma, 1 sarcoma, 1 seminoma, 1 choriocarcinoma | Liver & lung: 3  Unknown: 1 |
|  |  | 1 neck sarcoma | Liver & lung: 1 |
|  |  | Pelvis: 1 sarcoma, 1 primitive neuroectodermal tumor | Lung: 1  Liver & bones: 1 |
|  |  | Unknown paracaval mass | Liver & lung: 1 |
|  |  | Neuroblastoma in the upper left hemithorax: 1 | Liver, lung & bones: 1 |
|  | Unknown: 7 (9.1%) | Adenocarcinoma: 5  Neuroendocrine carcinoma: 1  Unknown: 1 | Liver: 2  Liver & lung: 3  Liver & bones: 1  Other: 1 |
| Cohort, N = 9 | Lung: 3 (33.3%) | Small cell lung cancer: 2  Adenocarcinoma: 1 | NA |
|  | Stomach: 1 (11.1%) | Adenocarcinoma: 1 | NA |
|  | Colon: 1 (11.1%) | Adenocarcinoma: 1 | NA |
|  | Uterus (Endometrium): 1 (11.1%) | Adenocarcinoma: 1 | NA |
|  | Esophagus: 1 (11.1%) | Squamous carcinoma: 1 | NA |
|  | Other: 1 (11.1%) | Quadriceps myxoid liposarcoma: 1 | NA |
|  | Unknown: 1 (11.1%) | NA | NA |

**Table S5.** Characteristics of included participants and STLS outcomes per site of the primary tumor. Abbreviations: TLS, tumor lysis syndrome; STLS, spontaneous tumor lysis syndrome; N, number of individuals whose data on the outcome was available; CCI, Charlson Comorbidity Index; AKI, acute kidney injury; NR, not reported; BUN, blood urea nitrogen; SeCr, serum creatinine; LDH, lactate dehydrogenase; WBC, white blood cells; CB, Cairo-Bishop; CTLS, clinical tumor lysis syndrome; ALL, allopurinol; FEB, febuxostat; RASB, rasburicase; RRT, renal replacement therapy.

**(a)** Patient demographics, tumor characteristics, and clinical characteristics and complications of STLS.

| Primary site | N (%) | Age | Sex, male | CCI | Tumor size | AKI | Cardiac arrhythmia | New seizure | Symptomatic hypocalcemia |
| --- | --- | --- | --- | --- | --- | --- | --- | --- | --- |
| Lung | 15 (21.1%) | 68 (59, 74) | 10/15 | 8 (8, 9) | 6 (3.5, 10.5) | 13/15 | 0/15 | 1/15 | 1/15 |
| Skin | 3 (4.2%) | 59 (46, 62) | 3/3 | 7 (6, 8) | NR | 3/3 | 1/3 | 0/3 | 0/3 |
| Colon | 6 (8.5%) | 48.5 (47.0, 66.0) | 1/6 | 6.5 (6.0, 9.0) | 7 | 6/6 | 0/6 | 0/6 | 0/6 |
| Liver | 5 (7.0%) | 70 (59, 72) | 4/5 | 9 (7, 11) | 14 (13, 14.6) | 4/5 | 1/5 | 0/5 | 1/5 |
| Prostate | 3 (4.2%) | 69 (56, 73) | 3/3 | 8 (7, 11) | NR | 3/3 | 1/3 | 0/3 | 0/3 |
| Uterus (endometrium/myometrium) | 4 (5.6%) | 58.5 (45.5, 62.0) | 0/4 | 6.5 (5.5, 8.5) | 11.9 (3.5, 20.2) | 2/4 | 0/4 | 0/4 | 0/4 |
| Stomach | 4 (5.6%) | 56.5 (43.5, 70.5) | 4/4 | 7.5 (6.5, 9.5) | 8 (7, 9) | 4/4 | 0/4 | 0/4 | 0/4 |
| Breast | 3 (4.2%) | 51 (36, 62) | 0/3 | 6 (6,7) | 5.5 (4.0, 7.0) | 2/3 | 0/3 | 0/3 | 0/3 |
| Kidney | 3 (4.2%) | 85 (56, 88) | 2/3 | 10 (7, 13) | 9.8 (9.5, 10.0) | 2/3 | 0/3 | 0/3 | 0/3 |
| Ovary | 2 (2.8%) | 55.5 (49, 62) | 0/2 | 5 (4, 6) | NR | 2/2 | 0/2 | 0/2 | 0/2 |
| Pancreas | 2(2.8%) | 62 (56, 68) | 0/2 | 7.5 (7.0, 8.0) | 10 | 2/2 | 0/2 | 0/2 | 0/2 |
| Adrenal gland | 2 (2.8%) | 72 (64, 80) | 2/2 | 7 (6, 8) | 20 | 2/2 | 0/2 | 0/2 | 0/2 |
| Uterus-Cervix | 1 (1.4%) | 35 | 0/1 | 6 | NR | 1/1 | 0/1 | 0/1 | 0/1 |
| Gallbladder | 1 (1.4%) | 77 | 0/1 | 9 | NR | 1/1 | 0/1 | 0/1 | 0/1 |
| Esophagus | 1 (1.4%) | 61 | 1/1 | 9 | NR | 0/1 | 0/1 | 0/1 | 0/1 |
| Other | Retroperitoneal:  4 (5.6%) | 36.5 (23.0, 54.0) | 2/4 | 6.0 (3.0, 6.5) | 14.0 (11.4, 19.5) | 4/4 | 0/4 | 0/4 | 0/4 |
|  | Pelvis:  2 (2.8%) | 43.5 (22.0, 65.0) | 1/2 | 7 (6, 8) | 7.5 | 2/2 | 0/2 | 0/2 | 0/2 |
|  | Eyeball:  1 (1.4%) | 69 | 1/1 | 8 | NR | 0/1 | 0/1 | 0/1 | 0/1 |
|  | Neck:  1 (1.4%) | 27 | 0/1 | 6 | 6 | 0/1 | 0/1 | 0/1 | 0/1 |
|  | Paraaortic:  1 (1.4%) | 52 | 1/1 | 7.5 (7.0, 9.0) | 8 | 1/1 | 0/1 | 0/1 | 0/1 |
|  | Upper left hemithorax:  1 (1.4%) | 27 | 0/1 | 6 | 10.7 | 0/1 | 0/1 | 0/1 | 0/1 |
| Unknown | 6 (8.5%) | 62 (50, 66) | 3/6 | 7.5 (7.0, 9.0) | 4 | 5/6 | 0/6 | 0/6 | 0/6 |
| Total | 71 (100%) | 59.5 (49.0, 70.0) | 38/71 | 7 (6, 9) | 8.85 (6.0, 13.0) | 59/71 | 3/71 | 1/71 | 2/71 |

**(b)** Laboratory characteristics of STLS.

| Primary site | N (%) | BUN (mg/dl) | SeCr (mg/dl) | Uric acid (mg/dl) | Potassium (mmol/l) | Phosphorus (mg/dl) | Calcium (mg/dl) | Sodium (mmol/l) | WBC (/μl) | LDH (U/l) | CB clinical grade (on presentation) |
| --- | --- | --- | --- | --- | --- | --- | --- | --- | --- | --- | --- |
| Lung | 15 (21.1%) | 73.7 (63.5, 110.0) | 3.5 (1.8, 4.7) | 15.0 (12.8, 17.6) | 5.9 (5.6, 6.9) | 6.2 (4.7, 7.1) | 8.6 (8.2, 9.6) | 135 (131, 140) | 15000 (12300, 19800) | 1470 (913, 2720) | No CTLS: 2/15  1: 2/15  2: 6/15  3: 4/15  4: 1/15 |
| Skin | 3 (4.2%) | NR | 1.8 (1.6, 2.1) | 13.9 (12.5, 21.4) | 6.3 (6.2, 6.6) | 6.8 (4.4, 7.9) | 7.5 (6.8, 8.2) | NR | NR | 5200 (4962, 20223) | 1: 2/2  2: 0/2  3: 0/2  4: 0/2 |
| Colon | 6 (8.5%) | 86.5 (76.0, 97.0) | 3.7 (3.4, 6.4) | 21.7 (20.3, 23.9) | 5.9 (5.3, 6.5) | 6.8 (4.4, 8.6) | 8.2 (7.4, 8.2) | 124.5 (123.0, 126.0) | 16950 (9700, 33700) | 2304 (1689, 3071) | 1: 1/6  2: 2/6  3: 3/6  4: 0/6 |
| Liver | 5 (7.0%) | 74 (42, 111) | 2.8 (2.0, 3.9) | 17.0 (12.2, 21.5) | 5.7 (5.0, 6.4) | 5.6 (4.8, 6.3) | 8.4 (6.4, 10.6) | 133 (122, 152) | 61800 | 863.5 (703.0, 1024.0) | 1: 1/4  2: 1/4  3: 2/4  4: 0/4 |
| Prostate | 3 (4.2%) | 32.1 (10.3, 45.9) | 3.6 (2.0, 5.9) | 14.7 (9.3, 16.5) | 6.4 (5.7, 7.5) | 3.7 (3.0, 5.3) | 9.8 (9.6, 10.0) | 128 (121, 135) | 9400 | 4023 | 1: 0/3  2: 1/3  3: 2/3  4: 0/3 |
| Uterus (endometrium/myometrium) | 4 (5.6%) | 45 | 4.2 (2.7, 5.8) | 14.0 (11.3, 18.9) | 6.1 (5.8, 6.2)) | 7.2 (6.3, 8.5) | 7.2 (6.2, 8.7) | 125 | 31400 (30800, 32000) | 1791 (1111, 2471) | No CTLS: 1/4  1: 1/4  2: 1/4  3: 1/4  4: 0/4 |
| Stomach | 4 (5.6%) | 77.1 (51.3, 140.4) | 3.9 (2.5, 8.2) | 15.8 (11.7, 22.4) | 5.5 (5.3, 5.9) | 7.4 (6.9, 11.5) | 8.0 (6.9, 9.2) | 137.4 (134.0, 146.0) | 12730 (11660, 13800) | 1170.5 (460.5, 7848.5) | 1: 0/4  2: 2/4  3: 1/4  4: 1/4 |
| Breast | 3 (4.2%) | 54 | 1.4 (0.9, 1.5) | 13.4 (10.1, 13.4) | 7.1 (6.5, 7.6) | 5.0 (4.7, 6.0) | 8.9 (7.6, 10.1) | 122 | 20000 | 937 (509, 4899) | No CTLS: 2/3  1: 1/3  2: 0/3  3: 0/3  4: 0/3 |
| Kidney | 3 (4.2%) | 62.4 (58.8, 66.0) | 4.3 (4.0, 4.7) | 17.9 (11.2, 24.6) | 5.0 | 9.0 (7.4, 10.5) | 7.0 (4.7, 9.4) | NR | 20500 | 2373 | 1: 0/2  2: 0/2  3: 2/2  4: 0/2 |
| Ovary | 2 (2.8%) | 47 | 4.4 (3.0, 5.8) | 11.6 (8.1, 15.0) | 4.9 | 8.7 | NR | 132 | 14300 | NR | 1: 0/2  2: 1/2  3: 1/2  4: 0/2 |
| Pancreas | 2(2.8%) | NR | 6.8 (1.7, 12.0) | 15.6 (14.4, 16.7) | 6.7 (5.8, 7.5) | 9.3 (6.5, 12.0) | NR | NR | NR | 714.5 (379.0, 1050.0) | 1: 1/2  2: 0/2  3: 0/2  4: 1/2 |
| Adrenal gland | 2 (2.8%) | 69 | 3.3 (2.8, 3.8) | 16.45 (16.4, 16.5) | 6.3 (6.0, 6.6) | 6.4 (5.8, 6.9) | 8.1 (7.8, 8.4) | NR | 26580 | 4543.5 (864.0, 8223.0) | 1: 0/2  2: 1/2  3: 1/2  4: 0/2 |
| Uterus-Cervix | 1 (1.4%) | 81 | 2.4 | 14.6 | 6.1 | 7.9 | 6.9 | NR | NR | 702 | 1: 0/1  2: 1/1  3: 0/1  4: 0/1 |
| Gallbladder | 1 (1.4%) | NR | 4.4 | 17.8 | 6.2 | 9.0 | 8.4 | NR | NR | NR | 1: 0/1  2: 0/1  3: 1/1  4: 0/1 |
| Esophagus | 1 (1.4%) | NR | 1.0 | 8.7 | 4.7 | 4.7 | NR | NR | NR | 1141 | No CTLS: 1/1  1: 0/1  2: 0/1  3: 0/1  4: 0/1 |
| Other | Retroperitoneal:  4 (5.6%) | 95.5 (68.5, 148.5) | 4.9 (3.8, 6.3) | 20.9 (16.3, 25.0) | 7.3 (5.1, 8.5) | 9.9 (9.2, 15.8) | 7.6 (6.5, 7.7) | 127.5 (127.0, 128.0) | NR | 2660.0 (818.0, 8562.5) | 1: 0/4  2: 2/4  3: 1/4  4: 1/4 |
|  | Pelvis:  2 (2.8%) | 122.5 (49.0, 196.0) | 4.8 (1.7, 7.9) | 13.5 (11.4, 15.5) | 5.3 (4.5, 6.1) | 5.3 (4.9, 5.7) | 7.9 (7.5, 8.2) | 144 | 10000 | 1677 (1120, 2234) | 1: 1/2  2: 0/2  3: 1/2  4: 0/2 |
|  | Eyeball:  1 (1.4%) | 85.9 | 4.0 | 24.6 | 5.4 | 3.8 | 8.4 | 139 | NR | 992 | NR |
|  | Neck:  1 (1.4%) | ΝR | NR | NR | NR | NR | NR | NR | NR | NR | NR |
|  | Paraaortic:  1 (1.4%) | 84.1 | 4.2 | 21.9 | 7.9 | 7.1 | 5.1 | 120 | NR | 13400 | 1: 0/1  2: 1/1  3: 0/1  4: 0/1 |
|  | Upper left hemithorax:  1 (1.4%) | ΝR | NR | 13.1 | 5.4 | 4.9 | 10.3 | NR | NR | 847 | No CTLS: 1/1  1: 0/1  2: 0/1  3: 0/1  4: 0/1 |
| Unknown | 6 (8.5%) | 94.0 (59.5, 171.4) | 3.2 (1.8, 4.7) | 14.1 (10.4, 14.6) | 5.7 (5.0, 5.8) | 8.7 (6.2, 11.9) | 8.7 (8.3, 9.2) | 132 | 21000 (14700, 36000) | 1174 (899, 1449) | 1: 2/6  2: 1/6  3: 3/6  4: 0/6 |
| Total | 71 (100%) | 73.7 (54.0, 100.0) | 3.5 (2.1, 4.7) | 15.0 (12.8, 20.3) | 6.0 (0.9) | 6.8 (5.2, 8.6) | 8.2 (7.1, 9.4) | 132 (126, 139) | 16950 (12300, 30300) | 1449 (864, 3922) | No CTLS: 7/66  1: 12/66  2: 20/66  3: 23/66  4: 4/66 |

**(c)** Management of patients with STLS.

| Primary site | N (%) | RASB | ALL | FEB | ALL or FEB | ALL, FEB or RASB | ALL or FEB + RASB | ALL + RASB | FEB + RASB | Type of treatment | RRT |
| --- | --- | --- | --- | --- | --- | --- | --- | --- | --- | --- | --- |
| Lung | 15 (21.1%) | 8/14 | 7/14 | 0/14 | 7/14 | 10/14 | 5/14 | 5/14 | 0/14 | None: 4/14  ALL: 2/14  RASB: 3/14  ALL + RASB: 5/14  FEB + RASB: 0/14 | 4/14 |
| Skin | 3 (4.2%) | 2/3 | 1/3 | 0/3 | 1/3 | 2/3 | 1/3 | 1/3 | 0/3 | None: 1/3  ALL: 0/3  RASB: 1/3  ALL + RASB: 1/3  FEB + RASB: 0/3 | 2/3 |
| Colon | 6 (8.5%) | 4/6 | 4/6 | 1/6 | 5/6 | 6/6 | 3/6 | 2/6 | 1/6 | None: 0/6  ALL: 2/6  RASB: 1/6  ALL + RASB: 2/6  FEB + RASB: 1/6 | 3/5 |
| Liver | 5 (7.0%) | 1/4 | 1/4 | 0/4 | 1/4 | 2/4 | 0/4 | 0/4 | 0/4 | None: 2/4  ALL: 1/4  RASB: 1/4  ALL + RASB: 0/4  FEB + RASB: 0/4 | 1/4 |
| Prostate | 3 (4.2%) | 2/3 | 1/3 | 0/3 | 1/3 | 2/3 | 1/3 | 1/3 | 0/3 | None: 1/3  ALL: 0/3  RASB: 1/3  ALL + RASB: 1/3  FEB + RASB: 0/3 | 2/3 |
| Uterus (endometrium/myometrium) | 4 (5.6%) | 3/3 | 1/3 | 0/3 | 1/3 | 3/3 | 1/3 | 1/3 | 0/3 | None: 0/3  ALL: 0/3  RASB: 2/3  ALL + RASB: 1/3  FEB + RASB: 0/3 | 1/4 |
| Stomach | 4 (5.6%) | 0/4 | 2/4 | 0/4 | 2/4 | 2/4 | 0/4 | 0/4 | 0/4 | None: 2/4  ALL: 2/4  RASB: 0/4  ALL + RASB: 0/4  FEB + RASB: 0/4 | 2/4 |
| Breast | 3 (4.2%) | 1/3 | 3/3 | 0/3 | 3/3 | 3/3 | 1/3 | 1/3 | 0/3 | None: 0/3  ALL: 2/3  RASB: 0/3  ALL + RASB: 1/3  FEB + RASB: 0/3 | 0/3 |
| Kidney | 3 (4.2%) | NR | 1/1 | NR | 1/1 | 1/1 | NR | NR | NR | None: 0/1  ALL: 1/1  RASB: 0/1  ALL + RASB: 0/1  FEB + RASB: 0/1 | 1/2 |
| Ovary | 2 (2.8%) | 1/2 | 1/2 | 0/2 | 1/2 | 1/2 | 1/2 | 1/2 | 0/2 | None: 1/2  ALL: 0/2  RASB: 0/2  ALL + RASB: 1/2  FEB + RASB: 0/2 | 0/2 |
| Pancreas | 2(2.8%) | 2/2 | 1/2 | 0/2 | 1/2 | 2/2 | 1/2 | 1/2 | 0/2 | None: 0/2  ALL: 1/2  RASB: 1/2  ALL + RASB: 0/2  FEB + RASB: 0/2 | 0/2 |
| Adrenal gland | 2 (2.8%) | 1/2 | 1/2 | 0/2 | 1/2 | 2/2 | 0/2 | 0/2 | 0/2 | None: 0/2  ALL: 1/2  RASB: 1/2  ALL + RASB: 0/2  FEB + RASB: 0/2 | 1/2 |
| Uterus-Cervix | 1 (1.4%) | 0/1 | 1/1 | 0/1 | 1/1 | 1/1 | 0/1 | 0/1 | 0/1 | None: 0/1  ALL: 1/1  RASB: 0/1  ALL + RASB: 0/1  FEB + RASB: 0/1 | 0/1 |
| Gallbladder | 1 (1.4%) | 1/1 | 0/1 | 0/1 | 0/1 | 1/1 | 0/1 | 0/1 | 0/1 | None: 0/1  ALL: 0/1  RASB: 1/1  ALL + RASB: 0/1  FEB + RASB: 0/1 | 1/1 |
| Esophagus | 1 (1.4%) | 0/1 | 1/1 | 0/1 | 1/1 | 1/1 | 0/1 | 0/1 | 0/1 | None: 0/1  ALL: 1/1  RASB: 0/1  ALL + RASB: 0/1  FEB + RASB: 0/1 | 0/1 |
| Other | Retroperitoneal:  4 (5.6%) | 2/4 | 0/4 | 0/4 | 0/4 | 2/4 | 0/4 | 0/4 | 0/4 | None: 2/4  ALL: 0/4  RASB: 2/4  ALL + RASB: 0/4  FEB + RASB: 0/4 | 3/4 |
|  | Pelvis:  2 (2.8%) | 2/2 | 0/2 | 0/2 | 0/2 | 2/2 | 0/2 | 0/2 | 0/2 | None: 0/2  ALL: 0/2  RASB: 2/2  ALL + RASB: 0/2  FEB + RASB: 0/2 | 1/2 |
|  | Eyeball:  1 (1.4%) | 0/1 | 0/1 | 0/1 | 0/1 | 0/1 | 0/1 | 0/1 | 0/1 | None: 1/1  ALL: 0/1  RASB: 0/1  ALL + RASB: 0/1  FEB + RASB: 0/1 | 0/1 |
|  | Neck:  1 (1.4%) | 0/1 | 1/1 | 0/1 | 1/1 | 1/1 | 0/1 | 0/1 | 0/1 | None: 0/1  ALL: 0/1  RASB: 1/1  ALL + RASB: 0/1  FEB + RASB: 0/1 | 0/1 |
|  | Paraaortic:  1 (1.4%) | 0/1 | 0/1 | 0/1 | 0/1 | 0/1 | 0/1 | 0/1 | 0/1 | None: 0/1  ALL: 1/1  RASB: 0/1  ALL + RASB: 0/1  FEB + RASB: 0/1 | 1/1 |
|  | Upper left hemithorax:  1 (1.4%) | 0/1 | 1/1 | 0/1 | 1/1 | 1/1 | 0/1 | 0/1 | 0/1 | None: 0/1  ALL: 1/1  RASB: 0/1  ALL + RASB: 0/1  FEB + RASB: 0/1 | 0/1 |
| Unknown | 6 (8.5%) | 3/6 | 4/6 | 0/6 | 4/6 | 6/6 | 1/6 | 1/6 | 0/6 | None: 0/6  ALL: 3/6  RASB: 2/6  ALL + RASB: 1/6  FEB + RASB: 0/6 | 2/6 |
| Total | 71 (100%) | 33/65 | 32/66 | 1/65 | 33/66 | 51/66 | 15/65 | 14/65 | 1/65 | None: 15/66  ALL: 18/66  RASB: 18/66  ALL + RASB: 14/66  FEB + RASB: 1/66 | 25/67 |

**(d)** Prognosis of patients with STLS.

| Primary site | N (%) | All-cause death | Death due to TLS | Recurrence |
| --- | --- | --- | --- | --- |
| Lung | 15 (21.1%) | 10/13 | 7/13 | 0/15 |
| Skin | 3 (4.2%) | 3/3 | 3/3 | 0/3 |
| Colon | 6 (8.5%) | 5/6 | 4/6 | 0/6 |
| Liver | 5 (7.0%) | 3/4 | 3/4 | 0/4 |
| Prostate | 3 (4.2%) | 2/3 | 1/3 | 0/3 |
| Uterus (endometrium/myometrium) | 4 (5.6%) | 3/4 | 2/4 | 0/4 |
| Stomach | 4 (5.6%) | 3/4 | 1/4 | 0/4 |
| Breast | 3 (4.2%) | 2/2 | 0/2 | 2/3 |
| Kidney | 3 (4.2%) | 3/3 | 1/3 | 0/3 |
| Ovary | 2 (2.8%) | 0/2 | 0/2 | 0/2 |
| Pancreas | 2 (2.8%) | 2/2 | 1/2 | 0/2 |
| Adrenal gland | 2 (2.8%) | 1/1 | 1/1 | 0/2 |
| Uterus-Cervix | 1 (1.4%) | 1/1 | 1/1 | 0/1 |
| Gallbladder | 1 (1.4%) | 1/1 | 1/1 | 0/1 |
| Esophagus | 1 (1.4%) | 0/1 | 0/1 | 0/1 |
| Other 10 (14.1%) | Retroperitoneal:  4 (5.6%) | 3/4 | 2/4 | 0/4 |
|  | Pelvis:  2 (2.8%) | 0/1 | 0/1 | 0/2 |
|  | Eyeball:  1 (1.4%) | 1/1 | 1/1 | 0/1 |
|  | Neck:  1 (1.4%) | 1/1 | 1/1 | 0/1 |
|  | Paraaortic:  1 (1.4%) | 1/1 | 0/1 | 0/1 |
|  | Upper left hemithorax:  1 (1.4%) | 1/1 | 1/1 | 0/1 |
| Unknown | 6 (8.5%) | 6/6 | 5/6 | 0/6 |
| Total | 71 (100%) | 52/65 | 36/65 | 2/70 |

**Table S6.** Unified analyses on patients with solid tumors developing spontaneous tumor lysis syndrome (STLS). Characteristics according to death or no death related to the STLS and its complications. Cases developing after recent exposure to corticosteroids are also included. Continuous data are presented as mean ± SD or median (Q1, Q3). All laboratory measurements are on presentation. Abbreviations: N, number of individuals whose data on the outcome was available; OR, odds ratio; CI, confidence interval; STLS, spontaneous tumor lysis syndrome; NA, not applicable; LDH, lactate dehydrogenase; WBC, white blood cells; ALL, allopurinol; FEB, febuxostat; RASB, rasburicase; RRT, renal replacement therapy.

| **Factor** | **N** | **Overall** | **Death due to TLS** | **No death due to TLS** | **p-value** | **Univariate logistic regression, Crude OR (95% CI); p-value** |
| --- | --- | --- | --- | --- | --- | --- |
| Age | 70 | 59 (49, 69) | 59 (48, 69) | 57 (49, 65) | 0.767 | 1.00 (0.97, 1.03); p = 0.987 |
| Sex (female vs. male) | 71 | Female: 33/71  Male: 38/71 | Female: 17/33  Male: 23/38 | Female: 16/33  Male: 15/38 | 0.445 | 0.69 (0.27, 1.78); p = 0.446 |
| Charlson Comorbidity Index | 69 | 7 (6, 9) | 8 (6, 9) | 7 (6, 9) | 0.557 | 1.07 (0.85, 1.35); p = 0.545 |
| Diagnosis of the primary tumor along with STLS | 69 | Yes: 39/69  No: 30/69 | Yes: 20/39  No: 19/30 | Yes: 19/39  No: 11/30 | 0.317 | 0.61 (0.23, 1.61); p = 0.318 |
| Time between diagnosis of the primary tumor and STLS (days) | 59 | 6 (0, 60) | 6 (2, 32) | 4 (0, 240) | 0.577 | 1.00 (0.99, 1.00); p = 0.114 |
| Any treatment for primary tumor | 69 | Yes: 15/69  No: 54/69 | Yes: 8/15  No: 31/54 | Yes: 7/15  No: 23/54 | 0.778 | 0.85 (0.27, 2.67); p = 0.778 |
| Surgical treatment of the primary tumor | 69 | Yes: 9/69  No: 60/69 | Yes: 4/9  No: 35/60 | Yes: 5/9  No: 25/60 | 0.488 | 0.57 (0.14, 2.34); p = 0.437 |
| Stage | 70 | I: 1/70  II: 1/70  III: 5/70  IV: 63/70 | I: 0/1  II: 0/1  III: 1/5  IV: 38/63 | I: 1/1  II: 1/1  III: 4/5  IV: 25/63 | 0.079 | Ν/Α |
| Tumor size | 28 | 8.85 (6, 12) | 10 (6, 13) | 8 (4, 10.5) | 0.549 | 1.03 (0.89, 1.20); p = 0.676 |
| Metastasis | 70 | Yes: 63/70 (90%)  No: 7/70 (10%) | Yes: 38/63  No: 1/7 | Yes: 25/63  No: 6/7 | **0.039** | **9.12 (1.03, 80.38); p = 0.047** |
| Metastatic site(s) | 70 | Liver: 21/70  Lung: 5/70  Bones: 2/70  Liver & lungs: 12/70  Liver & bones: 13/70  Lung & bones: 1/70  Liver, lung & bones: 4/70  Metastatic but no liver, lung, or bones: 5/70  No metastasis: 7/70 | Liver: 16/21  Lung: 4/5  Bones: 0/2  Liver & lungs: 8/12  Liver & bones: 6/13  Lung & bones: 1/1  Liver, lung & bones: 2/4  Metastatic but no liver, lung, or bones: 1/5  No metastasis: 1/7 | Liver: 5/21  Lung: 1/5  Bones: 2/2  Liver & lungs: 4/12  Liver & bones: 7/13  Lung & bones: 0/1  Liver, lung & bones: 2/4  Metastatic but no liver, lung, or bones: 4/5  No metastasis: 6/7 | **0.022** | (with “no metastasis” as a reference):  **Liver: 19.20 (1.84, 199.94); p = 0.013**  **Lung: 24.00 (1.14, 505.20); p = 0.041**  **Liver & Lung: 12.00 (1.05, 136.79); p = 0.045**  Liver & Bones: 5.14 (0.48, 55.64); p = 0.178  Liver, lung & bones: 6.00 (0.34, 107.42); p = 0.224  Metastatic but no liver, lung, or bones: 1.50 (0.07, 31.57); p = 0.794 |
| Liver (as one of the metastatic sites) vs. No metastasis at all | 57 | Liver: 50/57  No metastasis: 7/57 | Liver: 32/50  No metastasis: 1/7 | Liver: 18/50  No metastasis: 6/7 | **0.034** | **10.67 (1.19, 95.72); p = 0.034** |
| Lung (as one of the metastatic sites) vs. No metastasis | 29 | Lung: 22/29  No metastasis: 7/29 | Lung: 15/22  No metastasis: 1/7 | Lung: 7/22  No metastasis: 6/7 | **0.026** | **12.86 (1.29, 128.14); p = 0.029** |
| Bones (as one of the metastatic sites) vs. No metastasis | 27 | Bones: 20/27  No metastasis: 7/27 | Bones: 9/20  No metastasis: 1/7 | Bones: 11/20  No metastasis: 6/7 | 0.204 | 4.91 (0.50, 48.62); p = 0.174 |
| Liver (as one of the metastatic sites) vs. No liver metastasis | 69 | Yes: 50/69  No: 19/69 | Yes: 32/50  No: 7/19 | Yes: 18/50  No: 12/19 | **0.042** | **3.05 (1.02, 9.12); p = 0.046** |
| Lung (as one of the metastatic sites) vs. No lung metastasis | 69 | Yes: 22/69  No: 47/69 | Yes: 15/22  No: 24/47 | Yes: 7/22  No: 23/47 | 0.185 | 2.05 (0.71, 5.95); p = 0.185 |
| Bones (as one of the metastatic sites) vs. No bone metastasis | 69 | Yes: 20/69  No: 49/69 | Yes: 9/20  No: 30/49 | Yes: 11/20  No: 19/49 | 0.217 | 0.52 (0.18, 1.48); p = 0.221 |
| Time between STLS diagnosis and metastasis (days) | 59 | 0 (0, 6) | 0 (0, 14) | 0 (0, 0) | 0.235 | 0.998 (0.994, 1.002); p = 0.416 |
| Treatment of metastasis | 62 | Yes: 2/62  No: 60/62 | Yes: 2/2  No: 34/60 | Yes: 0/2  No: 26/60 | 0.505 | Ν/Α |
| Surgical treatment of metastasis | 62 | Yes: 1/62  No: 61/62 | Yes: 1/1  No: 35/61 | Yes: 0/1  No: 26/61 | 1.000 | Ν/Α |
| Cairo Bishop clinical STLS severity on presentation | 66 | Mild or No Clinical STLS: 38/66  Severe Clinical STLS: 28/66 | Mild or No Clinical STLS: 21/38  Severe Clinical STLS: 15/28 | Mild or No Clinical STLS: 17/38  Severe Clinical STLS: 13/28 | 0.891 | 0.93 (0.35, 2.49); p = 0.891 |
| Cairo Bishop clinical STLS severity on presentation | 66 | Mild Clinical STLS: 38/66  Severe Clinical STLS: 28/66 | Mild Clinical STLS: 21/38  Severe Clinical STLS: 15/28 | Mild Clinical STLS: 17/38  Severe Clinical STLS: 13/28 | 0.891 | 0.93 (0.35, 2.49); p = 0.891 |
| Acute kidney injury | 71 | Yes: 60/71  No: 11/71 | Yes: 34/60  No: 6/11 | Yes: 26/60  No: 5/11 | 1.000 | 1.09 (0.30, 3.97); p = 0.896 |
| Cardiac arrhythmia | 71 | Yes: 4/71  No: 67/71 | Yes: 3/4  No: 37/67 | Yes: 1/4  No: 30/67 | 0.627 | 2.43 (0.24, 24.60); p = 0.451 |
| New-onset seizure | 71 | Yes: 1/71  No: 70/71 | Yes: 0/1  No: 40/70 | Yes: 1/1  No: 30/70 | 0.437 | N/A |
| Symptomatic hypocalcemia | 71 | Yes: 2/71  No: 69/71 | Yes: 1/2  No: 39/69 | Yes: 1/2  No: 30/69 | 1.000 | 0.77 (0.05, 12.81); p = 0.855 |
| Blood urea nitrogen (mg/dl) | 40 | 73.87 (51.5, 98) | 80 (66, 100.34) | 56.4 (45.92, 94) | 0.061 | 1.01 (0.99, 1.03); p = 0.132 |
| Serum creatinine (mg/dl) | 64 | 3.56 (2.10, 5.02) | 3.80 (2.10, 5.22) | 3.50 (1.97, 4.94) | 0.898 | 1.01 (0.81, 1.24); p = 0.961 |
| Uric acid (mg/dl) | 67 | 15.0 (12.8, 20.3) | 15.6 (14.1, 20.3) | 14.2 (11.2, 20.3) | 0.078 | 1.07 (0.97, 1.17); p = 0.185 |
| Potassium (mmol/l) | 61 | 5.98 ± 0.99 | 6.02 ± 0.81 | 5.91 ± 1.26 | 0.696 | 1.11 (0.66, 1.89); p = 0.690 |
| Phosphorus (mg/dl) | 66 | 7.00 (5.30, 8.99) | 7.10 (5.40, 9.20) | 6.85 (5.28, 8.63) | 0.650 | 1.04 (0.91, 1.19); p = 0.574 |
| Calcium (mg/dl) | 55 | 8.23 ± 1.61 | 8.28 ± 1.42 | 8.16 ± 1.88 | 0.792 | 1.05 (0.75, 1.47); p = 0.787 |
| Sodium (mmol/l) | 26 | 132 (126, 139) | 130 (124.5, 133) | 134.5 (127, 140) | 0.157 | 0.93 (0.83, 1.03); p = 0.159 |
| LDH (U/l) | 46 | 1579.5 (899, 4055) | 1932.5 (1004, 3702.5) | 1367.5 (626, 4445) | 0.644 | 0.99 (0.99, 1.00); p = 0.626 |
| WBC (/μl) | 21 | 16950 (13800, 30300) | 20500 (16000, 32000) | 14050 (11660, 30300) | 0.121 | 1.00 (0.99, 1.00); p = 0.257 |
| RASB vs No RASB | 67 | Yes: 34/67  No: 33/67 | Yes: 22/34  No: 17/33 | Yes: 12/34  No: 16/33 | 0.274 | 1.73 (0.65, 4.60); p = 0.275 |
| ALL vs No ALL | 68 | Yes: 32/68  No: 36/68 | Yes: 15/32  No: 24/36 | Yes: 17/32  No: 12/36 | 0.100 | 0.44 (0.17, 1.18); p = 0.102 |
| FEB vs No FEB | 67 | Yes: 1/67  No: 66/67 | Yes: 0/1  No: 39/66 | Yes: 1/1  No: 27/66 | 0.418 | N/A |
| At least 1 of ALL/FEB vs No ALL/FEB | 68 | Yes: 33/68  No: 35/68 | Yes: 15/33  No: 24/35 | Yes: 18/33  No: 11/35 | 0.054 | 0.38 (0.14, 1.03); p = 0.057 |
| At least 1 of ALL/FEB/RASB vs No ALL/FEB/RASB | 68 | Yes: 53/68  No: 15/68 | Yes: 32/53  No: 7/15 | Yes: 21/53  No: 8/15 | 0.343 | 1.74 (0.55, 5.52); p = 0.346 |
| ALL/FEB + RASB vs No ALL/FEB + RASB | 67 | Yes: 14/67  No: 53/67 | Yes: 5/14  No: 34/53 | Yes: 9/14  No: 19/53 | 0.055 | 0.31 (0.09, 1.06); p = 0.062 |
| ALL + RASB vs No ALL + RASB | 67 | Yes: 13/67  No: 54/67 | Yes: 5/13  No: 34/54 | Yes: 8/13  No: 20/54 | 0.108 | 0.37 (0.11, 1.28); p = 0.116 |
| FEB + RASB vs No FEB + RASB | 67 | Yes: 1/67  No: 66/67 | Yes: 0/1  No: 39/66 | Yes: 1/1  No: 27/66 | 0.418 | N/A |
| Urate-lowering treatment type | 68 | None: 15/68  ALL: 19/68  RASB: 20/68  ALL + RASB: 13/68  FEB + RASB: 1/68 | None: 7/15  ALL: 10/19  RASB: 17/20  ALL + RASB: 5/13  FEB + RASB: 0/1 | None: 8/15  ALL: 9/19  RASB: 3/20  ALL + RASB: 8/13  FEB + RASB: 1/1 | **0.020** | See breakdown |
| ALL vs. None | 34 | ALL: 19/34  None: 15/34 | ALL: 10/19  None: 7/15 | ALL: 9/19  None: 8/15 | 0.730 | 1.27 (0.33, 4.93); p = 0.730 |
| RASB vs None | 35 | RASB: 20/35  None: 15/35 | RASB: 17/20  None: 7/15 | RASB: 3/20  None: 8/15 | **0.027** | **6.48 (1.32, 31.83); p = 0.021** |
| ALL + RASB vs None | 28 | ALL + RASB: 13/28  None: 15/28 | ALL + RASB: 5/13  None: 7/15 | ALL + RASB: 8/13  None: 8/15 | 0.662 | 0.71 (0.16, 3.23); p = 0.662 |
| FEB + RASB vs. None | 16 | FEB + RASB: 1/16  None: 15/16 | FEB + RASB: 0/1  None: 7/15 | FEB + RASB: 1/1  None: 8/15 | 1.000 | N/A |
| RASB vs. ALL | 39 | RASB: 20/39  ALL: 19/39 | RASB: 17/20  ALL: 10/19 | RASB: 3/20  ALL: 9/19 | **0.029** | **5.10 (1.11, 23.37); p = 0.036** |
| ALL + RASB vs. ALL | 32 | ALL + RASB: 13/32  ALL: 19/32 | ALL + RASB: 5/13  ALL: 10/19 | ALL + RASB: 8/13  ALL: 9/19 | 0.430 | 0.56 (0.13, 2.36); p = 0.432 |
| FEB + RASB vs. ALL | 20 | FEB + RASB: 1/20  ALL: 19/20 | FEB + RASB: 0/1  ALL: 10/19 | FEB + RASB: 1/1  ALL: 9/19 | 1.000 | N/A |
| ALL + RASB vs. RASB | 33 | ALL + RASB: 13/33  RASB: 20/33 | ALL + RASB: 5/13  RASB: 17/20 | ALL + RASB: 8/13  RASB: 3/20 | **0.009** | **0.11 (0.02, 0.58); p = 0.009** |
| FEB + RASB vs. RASB | 21 | FEB + RASB: 1/21  RASB: 20/21 | FEB + RASB: 0/1  RASB: 17/20 | FEB + RASB: 1/1  RASB: 3/20 | 0.190 | N/A |
| FEB + RASB vs. ALL + RASB | 14 | FEB + RASB: 1/14  ALL + RASB: 13/14 | FEB + RASB: 0/1  ALL + RASB: 5/13 | FEB + RASB: 1/1  ALL + RASB: 8/13 | 1.000 | N/A |
| Rehydration | 61 | Yes: 57/61  No: 4/61 | Yes: 33/57  No: 3/4 | Yes: 24/57  No: 1/4 | 0.638 | 0.46 (0.04, 4.68); p = 0.510 |
| Insulin | 66 | Yes: 9/66  No: 57/66 | Yes: 6/9  No: 32/57 | Yes: 3/9  No: 25/57 | 0.722 | 1.56 (0.36, 6.87); p = 0.555 |
| Calcium gluconate | 66 | Yes: 4/66  No: 62/66 | Yes: 4/4  No: 34/62 | Yes: 0/4  No: 28/62 | 0.131 | N/A |
| Hemodialysis | 68 | Yes: 27/68  No: 41/68 | Yes: 15/27  No: 23/41 | Yes: 12/27  No: 18/41 | 0.965 | 0.98 (0.37, 2.60); p = 0.965 |

**Table S7.** Unified analyses on patients with solid tumors developing STLS. Characteristics of patients according to whether they needed renal replacement therapy (RRT) or not. Cases developing after recent exposure to corticosteroids are also included. Continuous data are presented as mean ± SD or median (Q1, Q3).

| **Factor** | **N** | **Overall** | **Need for RRT** | **No Need for RRT** | **p-value** | **Univariate logistic regression, Crude OR (95% CI); p-value** |
| --- | --- | --- | --- | --- | --- | --- |
| Age | 72 | 59 (49, 69) | 59 (49, 69) | 59 (49, 69) | 0.756 | 1.01 (0.98, 1.04); p = 0.501 |
| Sex (female vs. male) | 73 | Female: 32/73  Male: 41/73 | Female: 12/32  Male: 17/41 | Female: 20/32  Male: 24/41 | 0.731 | 0.85 (0.33, 2.19); p = 0.731 |
| Charlson Comorbidity Index | 71 | 7 (6, 8) | 7 (6, 8) | 7 (6, 9) | 0.617 | 0.90 (0.70, 1.16); p = 0.409 |
| Diagnosis of the primary tumor along with STLS | 71 | Yes: 38/71  No: 33/71 | Yes: 14/38  No: 14/33 | Yes: 24/38  No: 19/33 | 0.631 | 0.79 (0.30, 2.06); p = 0.631 |
| Time between diagnosis of the primary tumor and STLS (days) | 60 | 5 (0, 47) | 9.5 (2.5, 77) | 3 (0, 45) | 0.209 | 0.999 (0.997, 1.001); p = 0.708 |
| Any treatment for primary tumor | 70 | Yes: 14/70  No: 56/70 | Yes: 6/14  No: 21/56 | Yes: 8/14  No: 35/56 | 0.713 | 1.25 (0.38, 4.10); p = 0.713 |
| Surgical treatment of the primary tumor | 71 | Yes: 8/71  No: 63/71 | Yes: 4/8  No: 23/63 | Yes: 4/8  No: 40/63 | 0.469 | 1.74 (0.40, 7.62); p = 0.463 |
| Stage | 71 | I: 1/71  II: 1/71  III: 5/71  IV: 64/71 | I: 0/1  II: 0/1  III: 3/5  IV: 26/64 | I: 1/1  II: 1/1  III: 2/5  IV: 38/64 | 0.876 | Ν/Α |
| Tumor size | 29 | 8.7 (6, 13) | 8 (4, 14) | 9.5 (6, 13) | 0.770 | 0.99 (0.86, 1.14); p = 0.905 |
| Metastasis | 72 | Yes: 64/72  No: 8/72 | Yes: 26/64  No: 3/8 | Yes: 38/64  No: 5/8 | 1.000 | 1.14 (0.25, 5.19); p = 0.865 |
| Metastatic site(s) | 71 | Liver: 21/71  Lung: 5/71  Bones: 3/71  Liver & lungs: 13/71  Liver & bones: 12/71  Lung & bones: 1/71  Liver, lung & bones: 4/71  Metastatic but no liver, lung, or bones: 5/71  No metastasis: 8/71 | Liver: 16/21  Lung: 4/5  Bones: 0/2  Liver & lungs: 8/12  Liver & bones: 6/13  Lung & bones: 1/1  Liver, lung & bones: 2/4  Metastatic but no liver, lung, or bones: 1/5  No metastasis: 1/7 | Liver: 5/21  Lung: 1/5  Bones: 2/2  Liver & lungs: 4/12  Liver & bones: 7/13  Lung & bones: 0/1  Liver, lung & bones: 2/4  Metastatic but no liver, lung, or bones: 4/5  No metastasis: 6/7 | 0.810 | (with “no metastasis” as reference):  Liver: 0.71 (0.13, 3.99); p = 0.702  Lung: 1.11 (0.11, 10.99); p = 0.928  Bones: 3.33 (0.20, 54.53); p = 0.398  Liver & Lung: 1.94 (0.32, 11.76); p = 0.469  Liver & Bones: 0.83 (0.13, 5.40); p = 0.848  Liver, lung & bones: 0.56 (0.04, 8.09); p = 0.667  Metastatic but no liver, lung, or bones: 2.50 (0.25, 24.72); p = 0.433 |
| Liver (as one of the metastatic sites) vs. No metastasis at all | 57 | Liver: 49/57  No metastasis: 8/57 | Liver: 18/49  No metastasis: 3/8 | Liver: 31/49  No metastasis: 5/8 | 1.000 | 0.97 (0.21, 4.54); p = 0.967 |
| Lung (as one of the metastatic sites) vs. No metastasis | 31 | Lung: 23/31  No metastasis: 8/31 | Lung: 10/23  No metastasis: 3/8 | Lung: 13/23  No metastasis: 5/8 | 1.000 | 1.28 (0.25, 6.69); p = 0.768 |
| Bones (as one of the metastatic sites) vs. No metastasis | 28 | Bones: 20/28  No metastasis: 8/28 | Bones: 7/20  No metastasis: 3/8 | Bones: 13/20  No metastasis: 5/8 | 1.000 | 0.90 (0.16, 4.92); p = 0.901 |
| Liver (as one of the metastatic sites) vs. No liver metastasis | 70 | Yes: 49/70  No: 21/70 | Yes: 18/49  No: 9/21 | Yes: 31/49  No: 12/21 | 0.630 | 0.77 (0.27, 2.19); p = 0.630 |
| Lung (as one of the metastatic sites) vs. No lung metastasis | 70 | Yes: 23/70  No: 47/70 | Yes: 10/23  No: 17/47 | Yes: 13/23  No: 30/47 | 0.555 | 1.36 (0.49, 3.75); p = 0.556 |
| Bones (as one of the metastatic sites) vs. No bone metastasis | 70 | Yes: 20/70  No: 50/70 | Yes: 7/20  No: 20/50 | Yes: 13/20  No: 30/50 | 0.698 | 0.81 (0.27, 2.38); p = 0.698 |
| Time between STLS diagnosis and metastasis (days) | 60 | 0 (0, 6) | 0 (0, 6.5) | 0 (0, 0) | 0.255 | 1.001 (0.997, 1.005); p = 0.372 |
| Treatment of metastasis | 62 | Yes: 2/62  No: 60/62 | Yes: 2/2  No: 21/60 | Yes: 0/2  No: 39/60 | 0.134 | Ν/Α |
| Surgical treatment of metastasis | 62 | Yes: 1/62  No: 61/62 | Yes: 1/1  No: 22/61 | Yes: 0/1  No: 39/61 | 0.371 | Ν/Α |
| Cairo Bishop clinical STLS severity on presentation | 68 | Mild or No Clinical STLS: 41/68  Severe Clinical STLS: 27/68 | Mild or No Clinical STLS: 9/41  Severe Clinical STLS: 18/27 | Mild or No Clinical STLS: 32/41  Severe Clinical STLS: 9/27 | **<0.001** | **7.11 (2.39, 21.14); p < 0.001** |
| Cairo Bishop clinical STLS severity on presentation | 68 | Mild Clinical STLS: 41/68  Severe Clinical STLS: 27/68 | Mild Clinical STLS: 9/41  Severe Clinical STLS: 18/27 | Mild Clinical STLS: 32/41  Severe Clinical STLS: 9/27 | **<0.001** | **7.11 (2.39, 21.14); p < 0.001** |
| Acute kidney injury | 73 | Yes: 61/73  No: 12/73 | Yes: 27/61  No: 2/12 | Yes: 34/61  No: 10/12 | 0.108 | 3.97 (0.80, 19.67); p = 0.091 |
| Cardiac arrhythmia | 73 | Yes: 4/73  No: 69/73 | Yes: 3/4  No: 26/69 | Yes: 1/4  No: 43/69 | 0.294 | 4.96 (0.49, 50.23); p = 0.175 |
| New-onset seizure | 73 | Yes: 1/73  No: 72/73 | Yes: 1/1  No: 28/72 | Yes: 0/1  No: 44/72 | 0.397 | N/A |
| Symptomatic hypocalcemia | 73 | Yes: 2/73  No: 71/73 | Yes: 1/2  No: 28/71 | Yes: 1/2  No: 43/71 | 1.000 | 1.54 (0.09, 25.57); p = 0.765 |
| Blood urea nitrogen (mg/dl) | 39 | 73.74 (49, 100) | 89.07 (52.5, 108.67) | 68.6 (49, 87.74) | 0.325 | 1.01 (0.99, 1.02); p = 0.333 |
| Serum creatinine (mg/dl) | 66 | 3.45 (2.07, 4.70) | 4.37 (3.40, 6.10) | 2.40 (1.70, 4.00) | **<0.001** | **1.47 (1.11, 1.94); p = 0.007** |
| Uric acid (mg/dl) | 69 | 15.0 (12.8, 20.1) | 16.65 (14.2, 22.35) | 14.4 (11.9, 16.5) | **0.006** | **1.18 (1.05, 1.32); p = 0.004** |
| Potassium (mmol/l) | 63 | 5.95 ± 0.98 | 6.02 ± 0.98 | 5.90 ± 1.00 | 0.628 | 1.14 (0.68, 1.91); p = 0.622 |
| Phosphorus (mg/dl) | 68 | 6.85 (5.20, 8.63) | 7.13 (5.20, 9.10) | 6.20 (5.20, 7.90) | 0.168 | 1.07 (0.94, 1.22); p = 0.324 |
| Calcium (mg/dl) | 55 | 8.32 ± 1.51 | 8.01 ± 1.66 | 8.53 ± 1.40 | 0.211 | 0.79 (0.54, 1.14); p = 0.210 |
| Sodium (mmol/l) | 25 | 132 (127, 139) | 133 (126, 144) | 131.5 (128, 139) | 0.784 | 1.02 (0.96, 1.09); p = 0.458 |
| LDH (U/l) | 49 | 1449 (899, 4023) | 2771 (1265, 10853) | 1050 (847, 2373) | **0.023** | **1.0001 (1.000008, 1.00030); p = 0.039** |
| WBC (/μl) | 22 | 18375 (13800, 30300) | 16950 (12300, 32000) | 19800 (14300, 21000) | 0.815 | 1.000 (0.9999, 1.0001); p = 0.365 |
| RASB vs No RASB | 69 | Yes: 35/69  No: 34/69 | Yes: 17/35  No: 11/34 | Yes: 18/35  No: 23/34 | 0.170 | 1.97 (0.74, 5.25); p = 0.172 |
| ALL vs No ALL | 70 | Yes: 35/70  No: 35/70 | Yes: 10/35  No: 19/35 | Yes: 25/35  No: 16/35 | **0.029** | **0.34 (0.13, 0.91); p = 0.031** |
| FEB vs No FEB | 69 | Yes: 1/69  No: 68/69 | Yes: 1/1  No: 27/68 | Yes: 0/1  No: 41/68 | 0.406 | N/A |
| At least 1 of ALL/FEB vs No ALL/FEB | 70 | Yes: 36/70  No: 34/70 | Yes: 11/36  No: 18/34 | Yes: 25/36  No: 16/34 | 0.057 | 0.39 (0.15, 1.04); p = 0.060 |
| At least 1 of ALL/FEB/RASB vs No ALL/FEB/RASB | 70 | Yes: 56/70  No: 14/70 | Yes: 23/56  No: 6/14 | Yes: 33/56  No: 8/14 | 0.903 | 0.93 (0.28, 3.04); p = 0.903 |
| ALL/FEB + RASB vs No ALL/FEB + RASB | 69 | Yes: 15/69  No: 54/69 | Yes: 5/15  No: 23/54 | Yes: 10/15  No: 31/54 | 0.518 | 0.67 (0.20, 2.24); p = 0.520 |
| ALL + RASB vs No ALL + RASB | 69 | Yes: 14/69  No: 55/69 | Yes: 4/14  No: 24/55 | Yes: 10/14  No: 31/55 | 0.305 | 0.52 (0.14, 1.85); p = 0.310 |
| FEB + RASB vs No FEB + RASB | 69 | Yes: 1/69  No: 68/69 | Yes: 1/1  No: 27/68 | Yes: 0/1  No: 41/68 | 0.406 | N/A |
| Urate-lowering treatment type | 70 | None: 14/70  ALL: 21/70  RASB: 20/70  ALL + RASB: 14/70  FEB + RASB: 1/70 | None: 6/14  ALL: 6/21  RASB: 12/20  ALL + RASB: 4/14  FEB + RASB: 1/1 | None: 8/14  ALL: 15/21  RASB: 8/20  ALL + RASB: 10/14  FEB + RASB: 0/1 | 0.148 | See breakdown |
| ALL vs. None | 35 | ALL: 21/35  None: 14/35 | ALL: 6/21  None: 6/14 | ALL: 15/21  None: 8/14 | 0.477 | 0.53 (0.13, 2.21); p = 0.386 |
| RASB vs None | 34 | RASB: 20/34  None: 14/34 | RASB: 12/20  None: 6/14 | RASB: 8/20  None: 8/14 | 0.487 | 2.00 (0.50, 8.00); p = 0.327 |
| ALL + RASB vs None | 28 | ALL + RASB: 14/28  None: 14/28 | ALL + RASB: 4/14  None: 6/14 | ALL + RASB: 10/14  None: 8/14 | 0.430 | 0.53 (0.11, 2.56); p = 0.433 |
| FEB + RASB vs. None | 15 | FEB + RASB: 1/15  None: 14/15 | FEB + RASB: 1/1  None: 6/14 | FEB + RASB: 0/1  None: 8/14 | 0.467 | N/A |
| RASB vs. ALL | 41 | RASB: 20/41  ALL: 21/41 | RASB: 12/20  ALL: 6/21 | RASB: 8/20  ALL: 15/21 | **0.043** | **3.75 (1.02, 13.80); p = 0.047** |
| ALL + RASB vs. ALL | 35 | ALL + RASB: 14/35  ALL: 21/35 | ALL + RASB: 4/14  ALL: 6/21 | ALL + RASB: 10/14  ALL: 15/21 | 1.000 | 1.00 (0.22, 4.47); p = 1.000 |
| FEB + RASB vs. ALL | 22 | FEB + RASB: 1/22  ALL: 21/22 | FEB + RASB: 1/1  ALL: 6/21 | FEB + RASB: 0/1  ALL: 15/21 | 0.318 | N/A |
| ALL + RASB vs. RASB | 34 | ALL + RASB: 14/34  RASB: 20/34 | ALL + RASB: 4/14  RASB: 12/20 | ALL + RASB: 10/14  RASB: 8/20 | 0.071 | 0.27 (0.06, 1.15); p = 0.077 |
| FEB + RASB vs. RASB | 21 | FEB + RASB: 1/21  RASB: 20/21 | FEB + RASB: 1/1  RASB: 12/20 | FEB + RASB: 0/1  RASB: 8/20 | 1.000 | N/A |
| FEB + RASB vs. ALL + RASB | 15 | FEB + RASB: 1/15  ALL + RASB: 14/15 | FEB + RASB: 1/1  ALL + RASB: 4/14 | FEB + RASB: 0/1  ALL + RASB: 10/14 | 0.333 | N/A |
| Rehydration | 63 | Yes: 60/63  No: 3/63 | Yes: 22/60  No: 3/3 | Yes:38/60  No: 0/3 | 0.058 | N/A |
| Insulin | 68 | Yes: 8/68  No: 60/68 | Yes: 6/8  No: 22/60 | Yes: 2/8  No: 38/60 | 0.057 | 5.18 (0.96, 27.92); p = 0.056 |
| Calcium gluconate | 68 | Yes: 3/68  No: 65/68 | Yes: 0/3  No: 28/65 | Yes: 3/3  No: 37/65 | 0.263 | N/A |

**Table S8.** Urate-lowering treatment [n (%)] per metastatic site of patients with solid tumors developing STLS (a) excluding and (b) including those with prior recent exposure to corticosteroids. Abbreviations: ALL, allopurinol; FEB, febuxostat; RASB, rasburicase.

**(a)** Fisher’s exact p-value is 0.403.

| Metastatic site | Type of treatment for hyperuricemia | | | | | |
| --- | --- | --- | --- | --- | --- | --- |
|  | None | ALL only | RASB only | ALL + RASB | FEB + RASB | Total |
| Liver | 5 (25%) | 4 (20%) | 6 (30%) | 5 (25%) | 0 (0%) | 20 |
| Lung | 2 (33.3%) | 0 (0%) | 4 (66.6%) | 0 (0%) | 0 (0%) | 6 |
| Bones | 1 (100%) | 0 (0%) | 0 (0%) | 0 (0%) | 0 (0%) | 1 |
| Other | 3 (60%) | 0 (0%) | 1 (20%) | 1 (20%) | 0 (0%) | 5 |
| No metastasis | 2 (25%) | 3 (37.5%) | 1 (12.5%) | 2 (25%) | 0 (0%) | 8 |
| Liver & lung | 2 (16.7%) | 2 (16.7%) | 3 (25%) | 4 (33.3%) | 1 (8.3%) | 12 |
| Liver & bones | 0 (0%) | 4 (50%) | 2 (25%) | 2 (25%) | 0 (0%) | 8 |
| Lung & bones | 0 (0%) | 1 (100%) | 0 (0%) | 0 (0%) | 0 (0%) | 1 |
| Liver, lung & bones | 0 (0%) | 2 (66.6%) | 1 (33.3%) | 0 (0%) | 0 (0%) | 3 |
| Total | 15 | 16 | 18 | 14 | 1 | 64 |

**(b)** Fisher’s exact p-value is 0.266.

| Metastatic site | Type of treatment for hyperuricemia | | | | | |
| --- | --- | --- | --- | --- | --- | --- |
|  | None | ALL only | RASB only | ALL + RASB | FEB + RASB | Total |
| Liver | 5 (25%) | 4 (20%) | 6 (30%) | 5 (25%) | 0 (0%) | 20 |
| Lung | 2 (33.3%) | 0 (0%) | 4 (66.6%) | 0 (0%) | 0 (0%) | 6 |
| Bones | 1 (50%) | 1 (50%) | 0 (0%) | 0 (0%) | 0 (0%) | 2 |
| Other | 3 (60%) | 0 (0%) | 1 (20%) | 1 (20%) | 0 (0%) | 5 |
| No metastasis | 2 (25%) | 3 (37.5%) | 1 (12.5%) | 2 (25%) | 0 (0%) | 8 |
| Liver & lung | 2 (15.4%) | 2 (15.4%) | 4 (30.8%) | 4 (30.8%) | 1 (7.6%) | 13 |
| Liver & bones | 0 (0%) | 5 (45.4%) | 4 (36.4%) | 2 (18.2%) | 0 (0%) | 11 |
| Lung & bones | 0 (0%) | 1 (100%) | 0 (0%) | 0 (0%) | 0 (0%) | 1 |
| Liver, lung & bones | 0 (0%) | 3 (75%) | 1 (25%) | 0 (0%) | 0 (0%) | 4 |
| Total | 15 | 19 | 21 | 14 | 1 | 70 |
